# The prognostic utility of the transcription factor SRF in docetaxel-resistant prostate cancer: in-vitro discovery and in-vivo validation

**DOI:** 10.1186/s12885-017-3100-4

**Published:** 2017-03-01

**Authors:** D. J. Lundon, A. Boland, M. Prencipe, G. Hurley, A O’Neill, E. Kay, S. T. Aherne, P. Doolan, S. F. Madden, M. Clynes, C. Morrissey, J. M. Fitzpatrick, R. W. Watson

**Affiliations:** 1 UCD School of Medicine, Conway Institute of Biomedical and Biomolecular Sciences, University College Dublin, Belfield, Dublin, Dublin 4 Ireland; 2 UCD School of Biomolecular and Biomedical Science, University College Dublin, Belfield, Dublin, Dublin 4 Ireland; 3 UCD School of Mathematical Sciences and Insight, University College Dublin, Belfield, Dublin, Dublin 4 Ireland; 4 National Institute for Cellular Biotechnology, Dublin City University, Dublin, Ireland Non-US/Non-Canadian Ireland; 5 Department of Pathology, Beaumont Hospital & Royal College of Surgeons in Ireland, Dublin, Ireland; 6 Department of Urology, University of Washington, Seattle, WA USA

**Keywords:** Prostate Cancer, Adenocarcinoma of prostate, Metastatic prostate cancer, Androgen-independent prostatic cancer, Docetaxel resistance, Anti-neoplastic agent resistance, Drug resistance, Personalised medicine, Translational oncology

## Abstract

**Background:**

Docetaxel based therapy is one of the first line chemotherapeutic agents for the treatment of metastatic castrate-resistant prostate cancer. However, one of the major obstacles in the treatment of these patients is docetaxel-resistance. Defining the mechanisms of resistance so as to inform subsequent treatment options and combinations represents a challenge for clinicians and scientists.

Previous work by our group has shown complex changes in pro and anti-apoptotic proteins in the development of resistance to docetaxel. Targeting these changes individually does not significantly impact on the resistant phenotype but understanding the central signalling pathways and transcription factors (TFs) which control these could represent a more appropriate therapeutic targeting approach.

**Methods:**

Using a number of docetaxel-resistant sublines of PC-3 cells, we have undertaken a transcriptomic analysis by expression microarray using the Affymetrix Human Gene 1.0 ST Array and in conjunction with bioinformatic analyses undertook to predict dysregulated TFs in docetaxel resistant prostate cancer. The clinical significance of this prediction was ascertained by performing immunohistochemical (IHC) analysis of an identified TF (SRF) in the metastatic sites from men who died of advanced CRPC. Investigation of the functional role of SRF was examined by manipulating SRF using SiRNA in a docetaxel-resistant PC-3 cell line model.

**Results:**

The transcription factors identified include serum response factor (SRF), nuclear factor kappa-B (NFκB), heat shock factor protein 1 (HSF1), testicular receptor 2 & 4 (TR2 &4), vitamin-D and retinoid x receptor (VDR-RXR) and oestrogen-receptor 1 (ESR1), which are predicted to be responsible for the differential gene expression observed in docetaxel-resistance. IHC analysis to quantify nuclear expression of the identified TF SRF correlates with both survival from date of bone metastasis (*p* = 0.003), survival from androgen independence (*p* = 0.00002), and overall survival from prostate cancer (*p* = 0.0044). Functional knockdown of SRF by siRNA demonstrated a reversal of apoptotic resistance to docetaxel treatment in the docetaxel-resistant PC-3 cell line model.

**Conclusions:**

Our results suggest that SRF could aid in treatment stratification of prostate cancer, and may also represent a therapeutic target in the treatment of men afflicted with advanced prostate cancer.

**Electronic supplementary material:**

The online version of this article (doi:10.1186/s12885-017-3100-4) contains supplementary material, which is available to authorized users.

## Background

Prostate cancer is the second most common cause of cancer and the sixth leading cause of cancer death amongst men worldwide [1]. Approximately 15% of men diagnosed with prostate cancer will die because of advanced metastatic disease; the majority of whom have castration resistant disease; and many of these will have received one or more treatment options [2]. Publications by Tannock et al. and Petrylak et al. demonstrated that docetaxel improved survival for men with metastatic castration resistant prostate cancer (mCRPC) [3, 4]. Despite new treatment options for prostate cancer, advanced disease still represents a challenge for treatment, and current treatment options for castration resistant disease offer limited survival advantage due to the development of resistance [5, 6].

Resistance to docetaxel is poorly understood, and may be caused by a number of mechanisms. These mechanisms include: (1) the fact that prostate tumours are slow-growing and are unlikely to respond to drugs that are S-phase dependent [7]. However, recent clinical trial data combining hormone ablation and docetaxel in hormone and chemo-naïve patients demonstrated an 18 month median overall survival (OS) advantage in patients with high volume prostate cancer [8]. (2) Reduced intra cellular concentrations of cytotoxic drugs as a result of alterations in drug transporters, particularly P-glycoprotein [9, 10]. (3) Tumour suppressor protein mutations, such as the loss of PTEN results in increased cellular proliferation and survival as well as activation of the phosphatidylinositol 3′-kinase (PI3K) signal transduction cascade [10, 11]. This is mediated through altered expression of survival factors that inhibit the apoptotic cell death pathway [10], mediated in part by survival signalling pathways such as the activation of AKT. (4) Alterations in β-tublin isotypes which exhibit different kinetics of microtubule formation particularly isotypes III and IV correlate with docetaxel resistance in vitro [12]. However the identification and manipulation of these multiple mechanisms of resistance represents a significant challenge and targeting individual proteins may have little clinical impact. More recently, O’Neill et al. undertook to characterise docetaxel resistance in prostate cancer cell lines [10]. This study highlighted a complex interplay between changes in the expression of both pro- and anti-apoptotic proteins which ultimately contributed to docetaxel resistance.

In the context of advanced, metastatic castration and docetaxel resistant prostate cancer, one or many of these pathways may be involved in its development. We hypothesised that by understanding the central signalling pathways and transcription factors (TFs) which govern multiple downstream genes we could identify key transcription factors, that when manipulated would alter docetaxel resistance. This study was undertaken to expand our understanding of the mechanisms of resistance to Docetaxel using our previously described PC-3 docetaxel resistant model [10].

Our objectives were to identify TFs which could account for this resistant phenotype in a model of docetaxel resistance, to validate these TFs in tissue from men who have died from docetaxel resistant mCRPC, and to evaluate if functional manipulation of such TFs could alter response to docetaxel therapy.

## Methods

### Cell lines

The human prostate cancer cell lines PC-3 were purchased from the American Type Culture Collection (ATCC CRL-1435). PC-3 resistant sublines (D8, and D12) and their corresponding age matched controls (Ag) were generated and maintained as previously described [10]. Briefly, these resistant sub-lines were generated by initially treating cells with increasing doses of docetaxel starting at 4 and 8nM respectively, escalating to 8 and 12 nm respectively with recovery periods between treatments of 2–3 weeks and treatments cycled over a period of 6 months. Their characteristics and IC50 have been previously published [10].

### RNA preparation and microarray analysis

Total RNA was isolated from the three PC-3 cell lines (aged matched control [Ag] and the 2 resistant sublines [D8, D12]) in four replicates; using methods previously described [6]. The Affymetrix Human Gene 1.0 ST Array containing 764,885 probe sets was used to perform gene expression profiling, and was used in accordance with the manufacturer’s instructions.

Gene expression values were calculated using the robust multichip average method [13] and data were quantile normalized using the Bioconductor package affy [14]. Differential gene expression lists were generated using the ebayes function of the limma package from Bioconductor [15]. The *P*-values were adjusted for multiple testing using the Benjamini and Hochberg method [16]. An adjusted *P*-value of <0.01 was considered significant. The choices of comparisons within the datasets were guided by the unsupervised co-inertia analysis (CIA) that is parental versus D8 and parental versus D12. The final gene list was determined by consistent overlap between these two comparisons.

### Co-Inertia Analysis (CIA)

The microarrays were analysed using a method for integrating gene expression data with known and predicted transcription factor binding site (TFBS) information [17]. This method uses CIA [13, 18] to combine two linked datasets, performing two simultaneous non-symmetric correspondence analyses and identifying the axes that are maximally co-variant. CIA is first applied in an unsupervised manner and then rerun in a supervised manner using between group analysis (BGA). This analysis was performed as previously described [6].

The final TF list was determined by overlap between these two ranked lists. All calculations were carried out using the MADE4 library [19] of the open source R package (http:// www.bioconductor.org).

### Transcription factor binding site information

The TFBS information, which is integrated with the gene expression data using CIA has been previously published [17]. It contains the TFBS information for 1,236 known and predicted TFBS conserved across human, mouse, rat and dog in the promoters of approximately 17,000 genes. This information was generated at four different position specific scoring matrix thresholds, 0.7, 0.75, 0.8, and 0.85 giving four gene/TFBS frequency tables. In the supervised CIA these thresholds are combined using the Rank Products method [20], giving a ranked list of TFs associated with docetaxel-resistance.

### Total cellular protein isolation and western blot analysis

Whole cell lysates were extracted from treated cells grown to 90% confluence on T75 flasks and 6-well plates as previously described [10]: Cells were washed in cold PBS (1100 rpm, 1 min, 4 °C in a microcentrifuge) and then re-suspended in Tris 10 mM, 60 mM KCl, NP-40, 1 mM EDTA, 1.0 mM DTT, 10 μl/ml Protease Inhibitor Cocktail (Sigma P8340)/1 ml of lysis buffer and 10 mM PMSF. Samples were then placed on ice for 10 mins and the cell lysate collected after centrifugation (13000 rpm 5 mins at 4 °C).

Total cellular protein was determined by means of the Bradford Assay Protein Detection Kit (Bio-Rad) as previously described [10]. Equal amounts of protein (50 μg) were subjected to SDS polyacrylamide gel electrophoresis on 8–12% gels before being trans-blotted onto Immobilin P (Millipore) membranes as previously published [10]. The following primary antibodies were used: anti-SRF (1:1,000, Santa Cruz) and ß-actin (1:5,000, Sigma–Aldrich). Densitometry values were calculated using ImageJ software [21].

### Small-Interfering RNA (siRNA) transfection

PC-3 parental and D12 cells were seeded in 6-well plates at a density of 250,000 cells per well. Twenty four hours later, cells were transfected with siGENOME SMART pool targeting serum response factor (SRF) (Dharmacon siGenome Human SRF #6722) or siControl siRNA (Dharmacon), at a final concentration of 20 nM, using Lipofectamine 2000 (Invitrogen). This commercially prepared product utilises a proprietary algorithm (SMARTselection algorithm™) to pool 5 SRF siRNA to alleviate off target effects and maintain effective silencing of SRF.

### Flow cytometric analysis of apoptosis

Apoptotic events were described as a percentage of total events with hypo-diploid DNA assessed by propidium iodide incorporation as previously described [10, 22]. Briefly, cells were harvested by trypsinisation, permeabilised with a hypotonic fluorochrome solution (50 mg/ml PI, 1 mM Tris, 0.1 mM EDTA, 3.4 mM sodium citrate and0.1% TritonX-100) and incubated for 10 min prior to analysis. Samples were run on a Beckman-Coulter FC-500 Cytometer. Ten thousand events were gated on PI intensity and analysed using CXP software (Beckman-Coulter).

### 3-(4,5)-dimethylthiazol-2-yl-2,5-diphenyltetrazolium bromide (MTT) assay cell viability assay

Cell viability was assessed by MTT cell staining as previously described [23]. Briefly, MTT (50 μl of a 5 mg/ml in PBS; Sigma-Aldrich) was added to each well and the cells were incubated in a CO_2_ incubator at 37 °C for 5 h. Following media removal, the MTT-formazan formed by metabolically viable cells was dissolved in 200 μl of DMSO (Sigma- Aldrich) and the absorbance was measured in a plate reader at 550 nm.

### Sample collection/tissue microarray construction

Human tissue microarrays were constructed consisting of 65 soft tissue metastases and 120 bone metastases from 42 patients with advanced prostate cancer as previously described [24]. This cohort had been recruited and work performed prior to the advent of novel anti-androgens (such as enzalutamide and abiraterone), however 50% of the cohort received radiotherapy and over 95% had received various combinations of therapies (chemotherapeutic agents/ immunotherapies). Samples were obtained from patients who died of mCRPC and who signed written informed consent for a rapid autopsy to be performed ideally within 2 h of death, under the aegis of the Prostate Cancer Donor Program at the University of Washington [24]. Cohort characteristics are outlined in Additional file 1: Table S1. Two replicate 1 mm cores of soft tissue metastases and bone metastases were taken from every patient where available [25]. The tissue microarrays were assembled using the Beecher Instruments Tissue-Arrayer™ (Beecher Instruments, Silver Spring, MD).

### Immunohistochemical (IHC) analysis

Immunohistochemical staining for SRF was performed using a microwave-induced antigen retrieval method as previously described [26]. De-waxed sections were immersed in a citric acid buffer and placed in a 700 W microwave oven at full power for 15 min. Using a standard avidin-biotin complex method (Vector Laboratories, Inc.), the sections were incubated with polyclonal rabbit (Santa Cruz Biotechnology, Inc. – 1:800 dilution) at 4 °C overnight. The colour reaction product was obtained with DAB and counterstained with Haematoxylin. Tonsil sections were used as positive controls. Prior to this study, the SRF antibody was subjected to western blot analysis using LNCaP and PC-3 prostate cancer cell lines which confirmed specificity for SRF [6].

### Scoring of SRF protein expression and statistical analysis

Nuclear immunoreactivity for SRF was assessed in soft tissue metastases and bone metastases by two independent observers (GOH) (EK). Unusable cores were found in the TMAs due to the tissue cores being missing, cancer necrosis, or insufficient cancer cells. These cores were excluded from the study. The cohort was divided using quartiles based on survival: [a] from diagnosis with prostate cancer [b] from diagnosis with CRPC and [c] from diagnosis with first bone metastasis; with the aim of extracting relatively homogenous subsections from an otherwise heterogeneous group. For the purpose of statistical analysis, immune-expression of the protein was graded according to the following scales: 0, no staining, 1, faint but clearly detectable staining in >10% of epithelial cells, 2, moderate staining in >10% of epithelial cells and 3, strong staining in >10% of epithelial cells.

The staining intensity of SRF in the nuclei of epithelial cells was then further divided into two groups: low expression (immunohistochemical score of 0 or 1) included those with negative or weak staining and high expression (immunohistochemical score of 2 or 3) included those with moderate or strong reactivity. Each individual’s SRF positivity was calculated by obtaining an average score of their sites of [i] bone metastasis, [ii] soft tissue metastasis [iii] both bone and soft tissue metastasis.

Chi square tests and Fisher exact tests were performed on 2X2 contingency tables using IBM SPSS 20 for Windows® to test the association of SRF immunohistochemical score (positive (2/3) and (negative (0/1)) with CRPC metastases type (bone metastases versus soft tissue metastases). Spearman’s rank correlation was performed using continuous variables, Kaplan-Meier curves plotted and logrank test performed using IBM SPSS 20 for Windows to test the relationship between SRF immunohistochemical score versus survival time from [a] diagnosis with PCa, [b] diagnosis with CRPC and [c] diagnosis with first bone metastasis. Multivariate analyses including other relevant clinical and pathological data available (age, primary and secondary Gleason score, number of bone metastases, number of soft tissue metastases, total number of metastases) was performed.

## Results

### Supervised CIA and differential gene expression analysis of PC-3 Cell line model of docetaxel resistance identifies TFs associated with docetaxel resistance

**Fig. 1:**
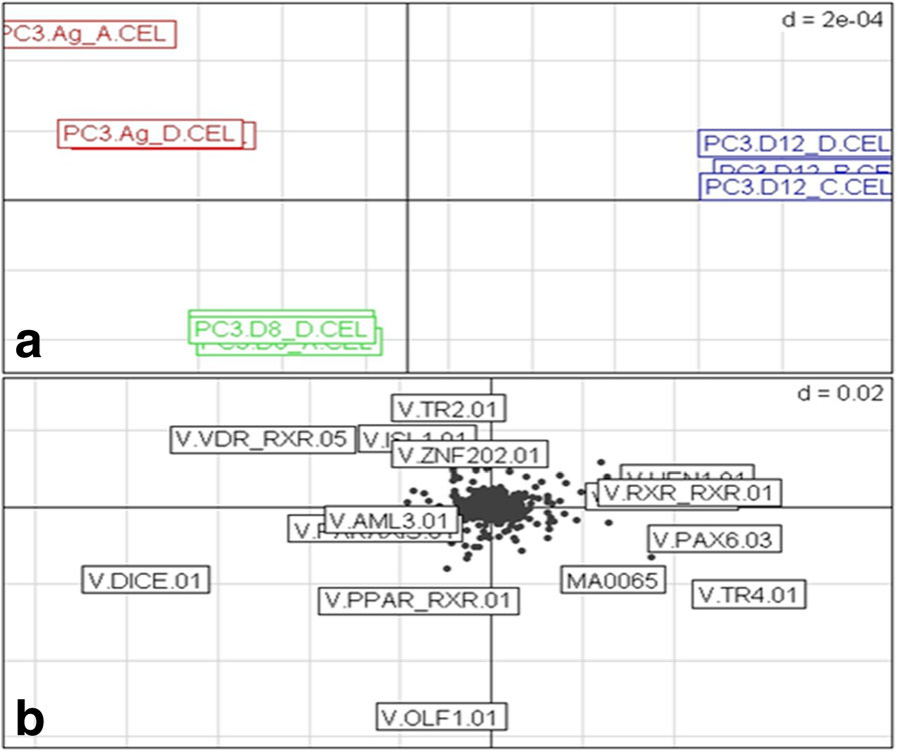
Unsupervised CIA of the PC-3 cell lines. A gene/transcription factor binding site (TFBS) frequency table produced with a position-specific scoring matrix (PSSM) threshold of 0.85 was used. **a**: The projection of the samples shows a clear separation between the parental and the two docetaxel resistant cell lines. **b**: The projection of the TFBS motifs is shown. Motifs that are in the same orientation as the docetaxel resistant cell lines in Fig. 1a are associated with docetaxel-resistance

To identify mechanisms of resistance to docetaxel within our dataset, all microarray data was analysed using CIA to integrate mRNA gene expression data and TFBS information in the promoters of the same genes. CIA was first applied in an unsupervised manner to the 12 arrays (four replicates for each cell line) and the associated TFBS/gene frequency tables to identify underlying trends in the data in each cell line. The aim of this analysis was to identify the TFs responsible for such trends and the differentially regulated genes they were predicted to target. An unsupervised CIA at the 0.85 PSSM thresholds (Fig. 1) was used for data exploration purposes. There was separation between the PC-3 parental cell line (Ag) and the docetaxel resistant subline (D8) along the vertical axis and between D12 and both the PC-3 parental cell line (Ag) and docetaxel resistant subline (D8) along the horizontal axis (Fig. 1a) and similarly for the transcription factor binding site (TFBS) motifs in the respective cell lines (Fig. 1b). These observations guided our choice of comparisons for both the supervised CIA and the differential gene expression analysis: Ag versus D8 versus D12.

To identify the TFBS specifically associated with docetaxel-resistance, we performed a supervised analysis of the data combining CIA and BGA using a methodology previously described [6]. This analysis returned three lists of motifs that were ranked based on the motif’s association with the docetaxel resistant cell lines. These lists of TFBS were then combined using the Rank Products method. Supervised CIA was used to analyse Ag versus D8, and Ag versus D12. The TFBS associated with docetaxel resistance were based on the overlap between these two comparisons.

The binary comparison between parental and D8 and parental and D12 were overlapped to identify genes which were differentially regulated in both cell lines. There were 716 probes up-regulated and 986 probes down-regulated between the two comparisons, indicating a tightly controlled experiment, and which corresponded to 301 distinct genes. Those genes, which were taken for further pathway analysis are listed in Additional file 2: Table S2, and the TFs that are predicted to target them are listed in Table 1. Close interplay between a sub-network of some of these TFs was identified and SRF was selected for further investigation.

**Table 1:** List of predicted transcription factors (TFs) associated with docetaxel-resistance

Symbol of predicted target	Description	RefSeq Accession	Log (Fold Change)	*P*-Value
NFKB2	Nuclear factor of Kapa Light Polypeptide gene enhancer in B-cells 2	NM 002502	−0.769367	0.000829
SRF	c-fos serum response element-binding transcription factor	NM_003131	0.830936	0.000984
TR2	nuclear receptor subfamily 2, group C, member 1	NM_003297.3	0.93892	0.000149
TR4	nuclear receptor subfamily 2, group C, member 2	NM_003298.3	−2.117639	1.54E-05
NR1H2	nuclear receptor subfamily 1, group H, member 2	NM_007121	2.086756	1.36E-05
BRN5	POU domain, class 6, transcription factor 1	NM_002702.3	1.090898	0.011998
PPAR_RXR	peroxisome proliferator-activated receptor alpha	NM_001001928.2	−0.570508	0.025654
ER	estrogen receptor 1	NM_000125.3	1.7681357	8.57E-06
NFE2L2	nuclear factor (erythroid-derived 2)-like 2	NM_001145412.2	0.415401	0.010718

Transcriptomic data was integrated with known and predicted transcription factor binding sites (TFBS) resulting in a list of transcription factors (TFs) associated with the differential gene expression observed with the transcriptomic profiling

### SRF expression is negatively correlated with docetaxel-resistance in metastatic castration resistant prostate cancer bone metastases

To evaluate SRF expression in mCRPC, we scored IHC staining of metastatic sites from 42 patients who died of CRPC. From this cohort, those who were treated with docetaxel were identified: 23 patients and 83 metastatic sites.

**Fig. 2:**
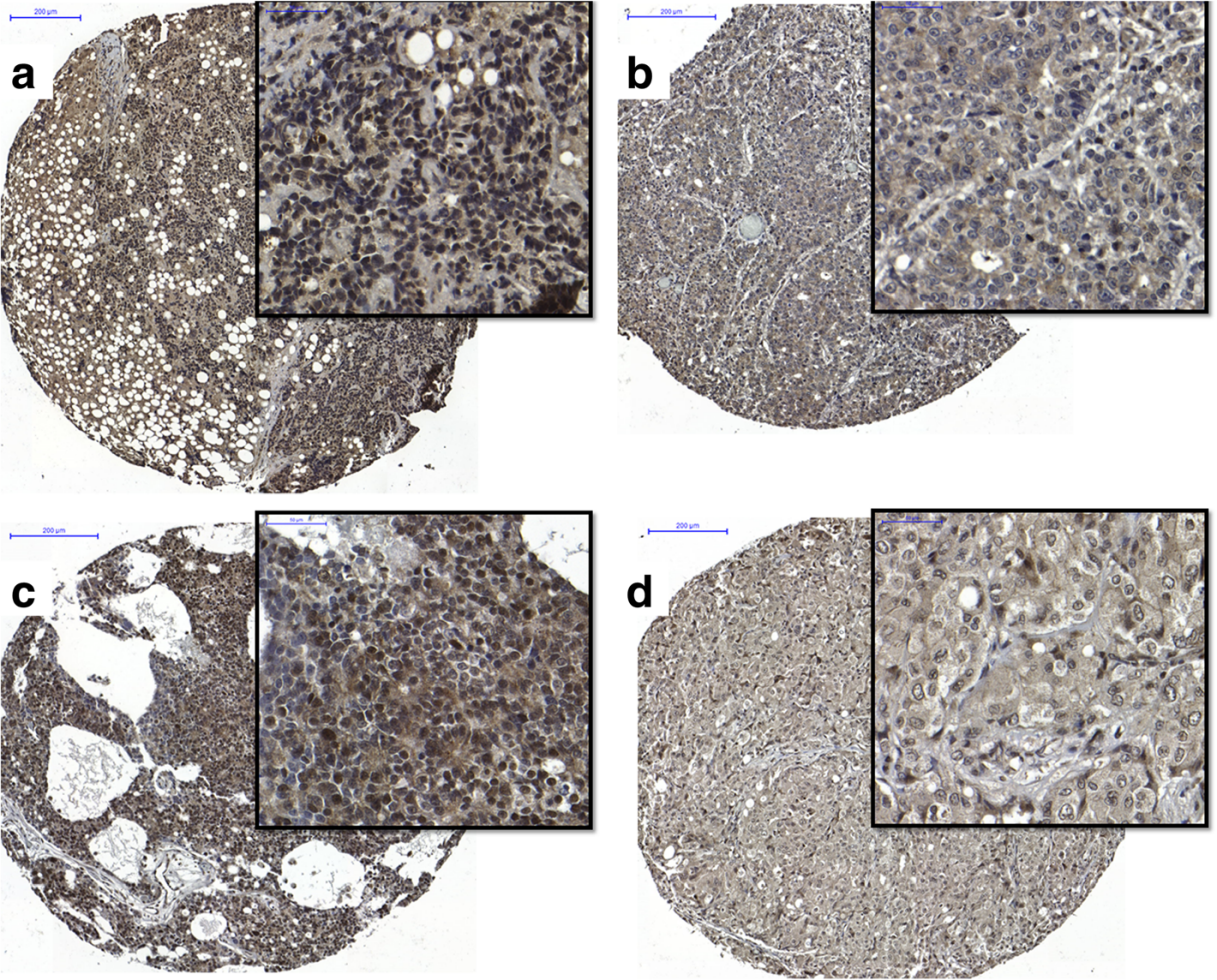
Representative images of serum response factor (SRF) protein expression assessed by immunohistochemistry on docetaxel resistant prostate cancer metastases; low power magnification of entire core and 40× magnification inset. Clockwise from top left **a**: bone metastasis demonstrating strong nuclear SRF expression, **b**: Bone metastasis demonstrating weak SRF nuclear expression, **d**: Soft tissue metastasis demonstrating weak SRF nuclear expression, **c**: Soft tissue metastasis demonstrating strong nuclear SRF expression. Images magnified × 40

Among 83 metastatic sites, 29 (35%) sites displayed positive nuclear SRF expression and 54 (65%) sites displayed negative SRF nuclear expression (see Fig. 2). The metastatic samples were then further divided into bone metastases versus soft tissue metastases. Out of a total of 52 bone metastatic sites, 20 (38%) sites had positive SRF nuclear expression and 32 (62%) sites displayed negative SRF nuclear expression. Out of a total of 31 soft tissue metastatic sites, 9 (29%) sites had positive SRF nuclear expression and 22 (71%) sites displayed negative SRF nuclear expression. Stepwise regression was performed including available clinical and pathological data were significant in the model.

### SRF expression in docetaxel resistant prostate cancer correlates with survival

**Fig. 3:**
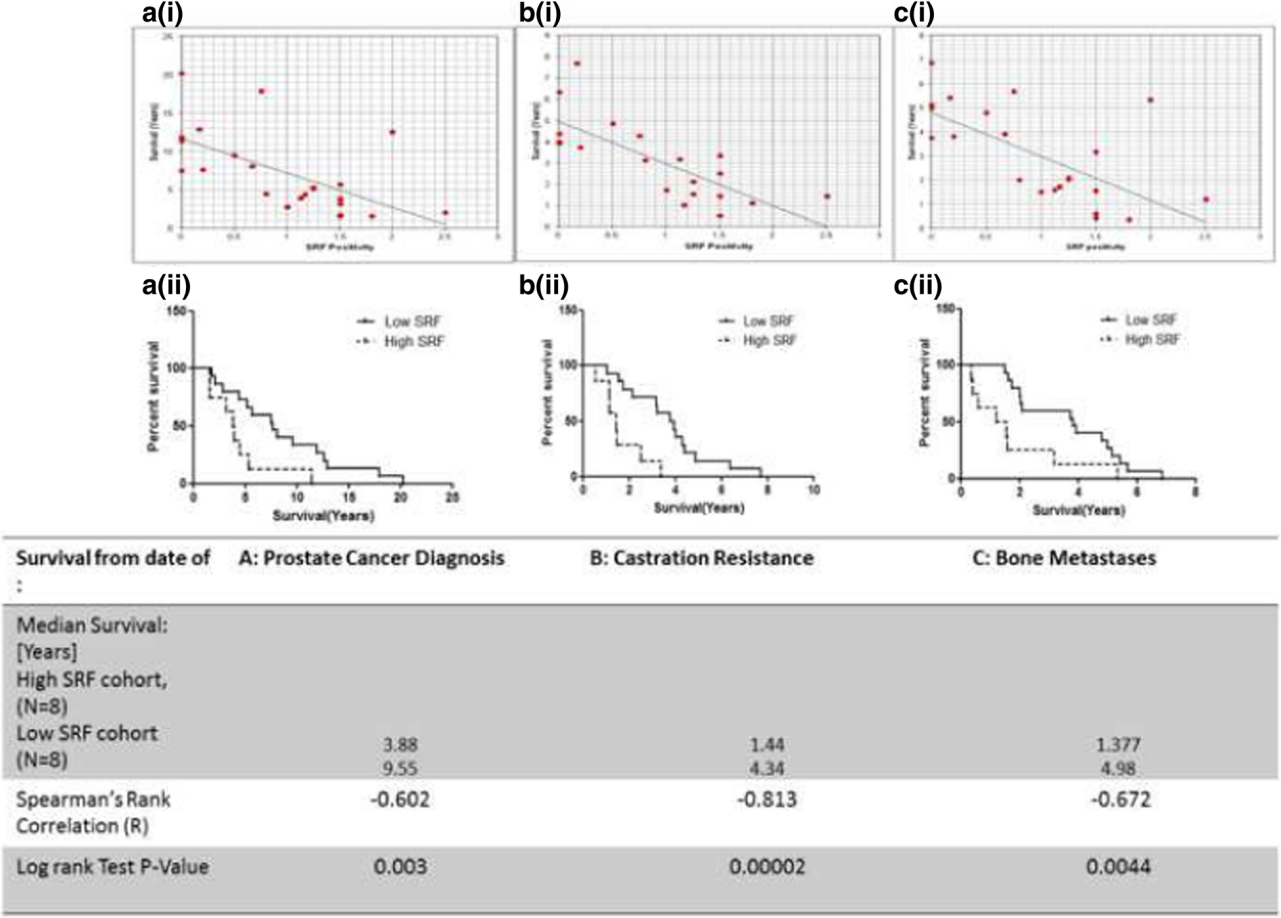
Correlation of SRF expression in bone metastases and survival: Tissues of docetaxel resistant prostate cancer bone metastases obtained at Rapid Autopsy were stained for SRF (*N* = 23). Time from (**a**) Prostate Cancer Diagnosis, (**b**) Castration Resistance and (**c**) Bone Metastases to death [Survival (Years)] was correlated with positivity of SRF in stained tissue samples. Correlation curves (i) and Kaplan-Meier curves (ii) at each of these time points respectively demonstrate the strong statistically significant negative correlation between nuclear expressivity of SRF and survival outcomes

A negative correlation was identified between SRF nuclear expression in bone metastases and survival from date of diagnosis with prostate cancer (Fig. 3a[i]; Spearman Rank Correlation −0.602, median difference in survival was 5.68 years), castration resistance (Fig. 3b[i]; Spearman Rank Correlation −0.813, median difference in survival was 2.89 years), and bone metastases (Fig. 3c[i]; Spearman Rank Correlation −0.672, median difference in survival was 3.6 years). Kaplan-Meier analysis was performed which confirmed SRF negative correlation from date of diagnosis with prostate cancer (Fig. 3a[ii]; Log-rank test, *P* = 0.003), castration resistance/ biochemical recurrence (Fig. 3b[ii]; Log-rank test, *P* = 0.00002), and bone metastases (Fig. 3C[ii]; Log-rank test, *P* = 0.0044). No association between SRF nuclear expression in soft tissue metastases and duration to death from diagnosis with prostate cancer (*P* = 0.744), diagnosis with CRPC (*P* = 0.292) or diagnosis with bone metastasis (*P* = 0.312) was observed.

In the portion of this cohort that did not receive docetaxel, median survival times from diagnosis with prostate cancer, castration resistance and bone metastasis were 4.95 years, 1.09 years and 2.22 years respectively, none of which were significantly different from the survival times in the docetaxel resistant cohort whose survival times from these time points were 5.33, 3.16 and 2.09 years respectively; (the respective p-values are 0.36, 0.26 and 0.28, denoting no significant difference in survival times between the docetaxel-resistant and docetaxel-naïve sub-cohorts).

When these sub-cohorts are further sub-divided by their expressivity of SRF in bone metastases (high SRF expressivity vs low SRF expressivity), as described above low SRF correlates with longer survival times from diagnosis, castration resistance and bone metastasis in the context of docetaxel resistance; however in the context of docetaxel naïve patients, SRF level does not correlate with survival times from these three clinically relevant time points (*p* values = 0.29, 0.30 and 0.38 respectively).

### Functional relevance of SRF in a docetaxel resistant model of advanced prostate cancer

#### Docetaxel treatment increases SRF transcriptional activity in docetaxel-resistant model

**Fig. 4:**
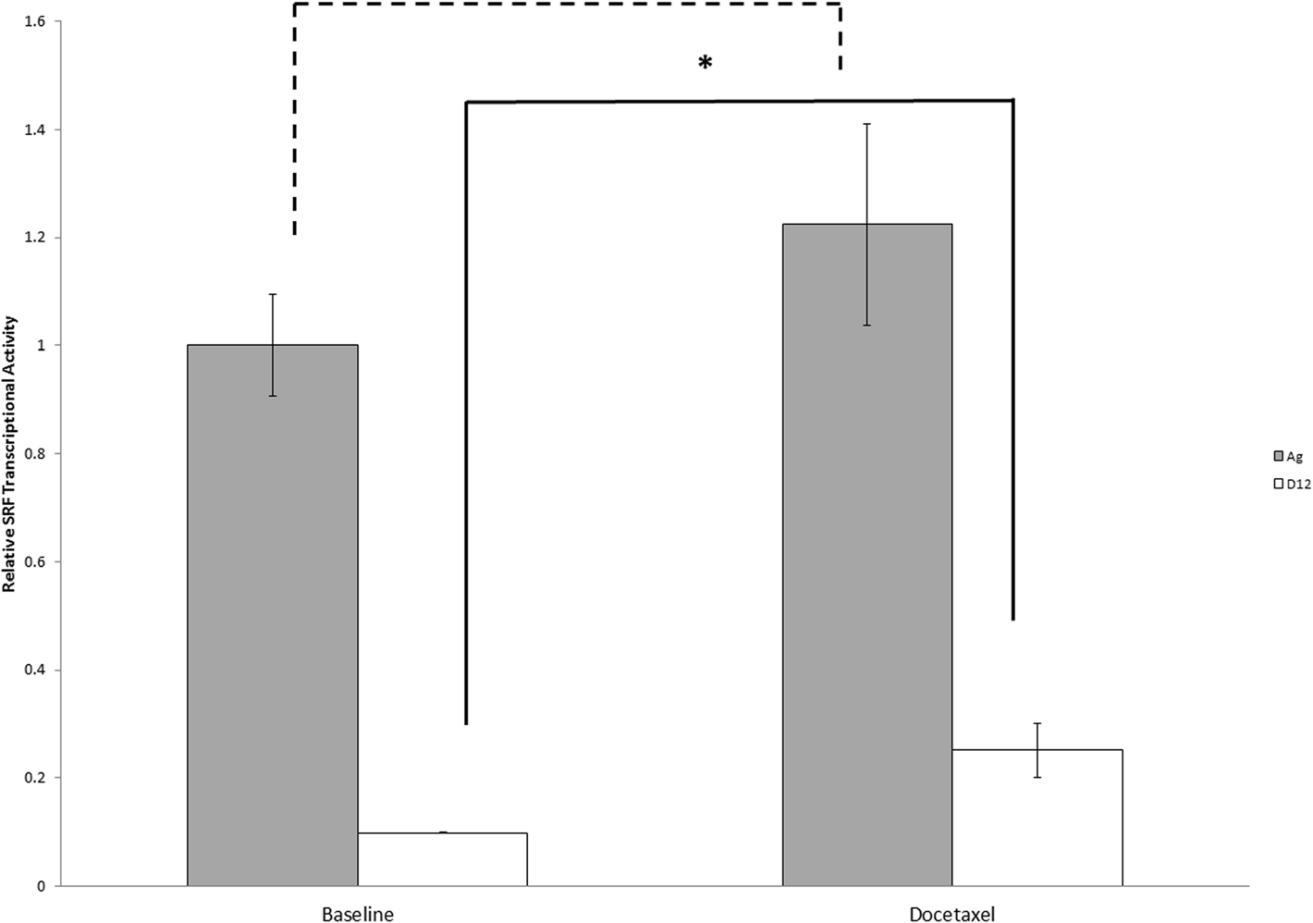
SRF transcriptional activity was assessed in Ag and PC3 docetaxel resistant (D12) cells which were seeded in 12-well plates at a density of 100,000 cells per well. The following day they were transiently transfected using a dual luciferase assay system, where the reporter construct is driven by SRF and tK renilla responsive elements. Twenty-four hours post-transfection, cells were treated with either 20 nM docetaxel or a similar volume of vehicle control for 6 h. Reporter gene activity was then measured by illuminometry, and relative SRF:tkRenilla transcriptional activity calculated. * = *p* < 0.05. No statistical difference between SRF transcriptional activity in PC3-Ag cells at baseline vs. treatment with docetaxel was observed (represented by the *dashed line*). (*n* = 3.)

To evaluate the functional role of SRF in the PC-3 model of docetaxel-resistance, we assessed transcriptional activity of SRF at baseline and following 48 h of treatment with docetaxel, in both a docetaxel-resistant subline (D12) and aged matched controls (Ag) (Fig. 3), using a dual-luciferase assay system. PC3-Ag cells demonstrated significantly greater SRF transcriptional activity than PC3-D12 cells at baseline. Following treatment with docetaxel, there was no increase in the relative SRF transcriptional activity in the PC3-Ag cells, but a greater than 2× increase in SRF transcriptional activity in the PC3-D12 cells (*p* = 0.009) (Fig. 4). This observation that SRF transcriptional activity is increased in response to docetaxel treatment in these resistant cells, but not in the docetaxel sensitive cells suggests that SRF transcriptional activation is a survival pathway in docetaxel resistance.

#### SRF knockdown (siRNA) re-sensitises resistant cells to docetaxel

**Fig. 5:**
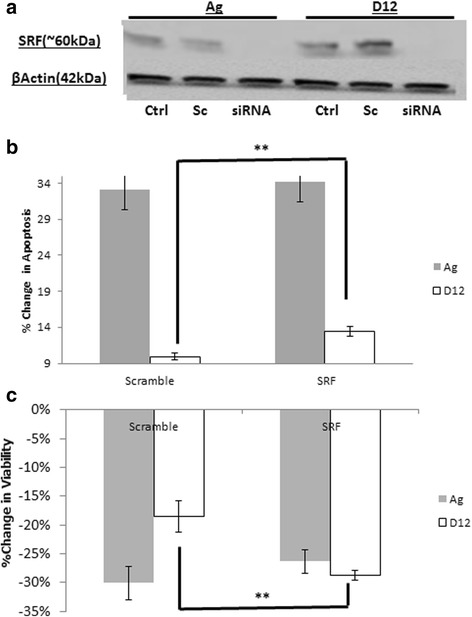
Functional Manipulation of SRF. **a** PC3-Age matched control (Ag) and PC3-docetaxel resistant (D12) cells for Western blotting analysis of SRF. β-actin was used as loading control. Fifty microgrammes of protein from untreated control (Ctrl), cells transfected with an empty vector; scramble control (Sc) and cells transfected with SRF siRNA knockdown (siRNA), were loaded into their respective wells. A representative image from three independent experiments is shown. SRF knockdown by siRNA was performed 48 h prior to treatment with 20 nM docetaxel for a further 48 h in 6 well plates seeded with ~100,000 cells per well of Ag and D12 cell lines respectively. **b**: Apoptosis was assessed using propidium iodide and flow cytometry (*n* = 3) and (**c**) Viability was assessed by MTT assay (*n* = 3). * = *p* < 0.05. ** = *p* < 0.01

To investigate if manipulation of SRF transcriptional activity in the resistant subline (PC3-D12) alters the sensitivity of these cells to docetaxel, 20nM SRF siRNA transfection was performed and cells allowed to recover for 48 h. Knockdown of SRF was confirmed at the protein level (Fig. 5a). Following knockdown, cells were treated with docetaxel [20nM] for 48 h. Cells were then assessed for apoptosis and viability. Flow cytometric analysis demonstrated no change in apoptosis in PC3-Ag cells but a significant increase in apoptosis in the PC3-D12 cells post-docetaxel treatment (*P* < 0.01) (Fig. 5b). Cell viability assessed by MTT assay similarly demonstrated no change in viability in the PC3-Ag whilst PC3-D12 cells demonstrated a significant reduction in viability (*p* < 0.01) (Fig. 5c).

## Discussion

Gene expression profiling has been shown to predict clinical outcomes of prostate cancer [27] but complex gene expression profiles are often difficult to manipulate. Targeting the TFs associated with this profile may represent a better therapeutic approach. This study predicted TFs associated with docetaxel-resistance based on transcriptomic data by utilising an innovative bioinformatics approach (CIA) and compared gene expression profiling of the PC3- Ag cells versus the docetaxel resistant cell lines D8 and D12. In line with recent transcriptomic studies by our group and others on castration-resistance [6, 28–31], analysis of our gene chip data showed gene expression changes in cellular processes relevant to cancer progression. These included cell proliferation, apoptosis, cell growth, survival and senescence and cell death with 375 unique genes differentially expressed between the parental Ag and docetaxel resistant sublines D8 and D12. The focus on upstream TFs regulating the transcriptomic profile rather than the gene expression offered the most novel insights: where transcriptomic data of docetaxel resistant cell lines was combined with a database of TFBS to identify TFs associated with docetaxel-resistance. The utilisation of this approach generated a list of 9 TFs (Table 1) predicted to be associated with docetaxel resistance in prostate cancer. Members of this list have previously been associated with prostate cancer, where decreased expression of ESR1 has been found to be particularly associated with hormone refractory disease [32], and PPARγ whose activity is regulated by direct binding of steroid and thyroid hormones, vitamins, lipid metabolites and xenobiotics and have been shown to participate in the development of the disease [33, 34].

Novel factors associated with docetaxel resistance in prostate cancer included: (1) SRF which is known to be involved with cancer development and progression and its role in castration resistance was previously outlined by our group [6]. (2) BRN5, a pou domain TF of which very little is known, and (3) TR2 and TR4; members of the orphan nuclear receptor family, for which activation or deactivation involves an intricate interplay of different structural classes of endogenous ligands such as the heterodimeric receptors that partner with the retinoid X receptor and bind retinoids and vitamin D [35]. In support of our findings, in recent months Chen et al. demonstrated that TR4 enhances the chemo-resistance of docetaxel in CRPC, and that it may serve as a biomarker to determine the prognosis of docetaxel-based therapy [36].

The dataset and TF list identified by our study represents a useful resource for future studies on docetaxel-resistance with valuable targets to be explored, as resistance is complex and the mechanisms underlying it multifarious [37]. For the purpose of validating this study we chose to further investigate the functional significance of SRF. SRF is expressed in mature soft tissues such as lung, liver and prostate and has been noted to be dysregulated in a number of malignant tissues such as prostate, breast, gastric and liver carcinoma [38–44]. In primary gastric cancers- high SRF correlates with a more invasive cancer phenotype and high SRF acts as an independent risk factor of short disease free survival [38]. SRF has been associated with prostate cancer development and progression [45–48], and our group have previously studied its role in the development of castration resistance [49]. SRF has also recently been associated with androgen receptor (AR) hypersensitivity; where a negative feedback loop exists between SRF expression and AR transcriptional activity in the setting of castrate-resistant prostate cancer [50]. This study gave us the opportunity to expand our understanding of SRF’s role in docetaxel resistance, in the context of AR negative and docetaxel resistant PC-3 cells, and clinical tissues from castrate and docetaxel resistant prostate cancer.

The treatment of men with mCRPC has seen a large number of changes since 2004. Prior to 2004, men who failed primary androgen deprivation were then treated palliatively. Since 2010 the therapeutic armamentarium has increased, but median survival of mCRPC in the post-docetaxel setting is 15-18months [51, 52]. This has led to calls for biomarkers of treatment response and a deeper understanding of the tumour heterogeneity and molecular biology underlying the disease [5]. Previous studies have demonstrated that SRF is associated with Gleason grade and extracapsular extension [46], poor post-operative outcome [45], and castration resistance [6]. To our knowledge, this study is the first to characterise the role of SRF in docetaxel-castration resistant prostate cancer. We found that nuclear tissue expression of SRF is significantly dysregulated in bone metastases of men with mCRPC in the post-docetaxel setting; such that low SRF expression is associated with significantly longer time to bone metastasis. Our research group and others have previously reported that SRF nuclear positivity is associated with higher Gleason score in primary prostate cancer tissues [46] and castrate-resistant TURPs [6] suggesting that SRF may play a role in prostate cancer progression. Additionally our group has demonstrated an association between SRF nuclear positivity and castration-resistant TURPs, with 95% of castrate-resistant TURPs showing nuclear positivity for SRF [6]. In our study of prostate cancer metastases to bone and soft tissue in men with advanced disease, approximately 40% displayed SRF nuclear positivity. In this cohort of men with mCDRPC, a negative association between SRF nuclear expression in bone metastases and survival from time of diagnosis with (1) prostate cancer (2) diagnosis with CRPC and (3) diagnosis with first bone metastasis was seen, which was independent from the number of metastatic sites. No significant association was noted between SRF and survival times in those men with mCRPC who had not been treated with Docetaxel. This finding demonstrates that with disease progression from localised prostate cancer, castration resistance and bone metastases; patients’ survival was inversely correlated with nuclear SRF expression in the context of docetaxel resistance.

Our group has also recently demonstrated that SRF has a negative association with the androgen receptor in CRPC and SRF is involved in the development of castration resistance [50]. In this cohort of men with mCRPC, the median difference in duration of androgen ablation between those subsequently classified as “high SRF and “low SRF” was 4.3 years (*p* = 0.000019). These findings suggest that those who have higher SRF are likely to have had more aggressive/adaptive disease, having evolved resistance to castration significantly sooner (by 4.3 years). Our data demonstrates a non-significant trend amongst those with SRF and duration of docetaxel therapy; with those with high SRF having received docetaxel for a shorter duration (median 0.166 years) compared to those with a low SRF (median duration 1.05 years).

This transition of SRF expression levels from primary to metastatic tissues, castration resistance and docetaxel therapy, amongst other factors, may explain the findings of a phase III randomised controlled trial. CHAARTED randomized men with newly diagnosed metastatic prostate cancer to ADT alone or ADT plus 6 cycles of docetaxel [8]. In this castration sensitive group, Sweeney et al. described a median OS of 57.6 months in the ADT plus docetaxel group, versus 44 months median OS in the ADT alone group (*p* = 0.003). This survival benefit contrasts sharply with docetaxel therapy in the castration resistant setting where median survival was 18.9 months in the docetaxel q 3 weekly group, versus 16.5 months median overall survival in the mitoxanthrone group (*p* = 0.009) [4]. Nuclear SRF expression is associated with castration resistance [6], and nuclear positivity is associated with shorter survival from castration resistance [26], and this study has demonstrated that high SRF expression after docetaxel therapy is correlated with a shorter survival. SRF and other factors likely represent a marker of disease progression; a common denominator or a waypoint in the pathway through which docetaxel and androgen ablation therapies exert their therapeutic effect in prostate cancer (so that men receiving combination therapy in CHAARTED who have progressive disease, are likely to express high levels of SRF in their primary tumour and bone metastases.

The finding that nuclear expression of SRF in soft tissue metastases does not correlate with survival from diagnosis with prostate cancer, castration resistance or first bone metastasis is likely due to a combination of factors including the heterogeneity of prostate cancer metastases, features unique to the respective microenvironments as opposed to just differential bioavailability of docetaxel in various tissue types. This distinction of microenvironmental factors from bioavailability in bone is made as Brubaker et al. have shown in in-vivo models of prostate cancer that docetaxel at a dose which effectively inhibits growth of subcutaneous tumours did not show any effect on the tumours in bone [53]. Meanwhile, Van Der Veldt et al. demonstrated adequate bioavailability of docetaxel in vertebrae in cancer patients, which was comparable to the bioavailability of docetaxel in lung tissues of these patients [54]. This differential effect of docetaxel in different tissue types, may in part be explained by SRF; SRF is associated with mesodermal formation; the embryonic germ layer from which bone and skeletal muscle is derived, in contrast with the endodermal origin of lung, liver and lymph nodes. The relationship of SRF to the origin of the tissues combined with our finding that high SRF in bone metastases is associated with shorter survival supports the role of SRF as a marker of docetaxel resistance, while the differential relationship between nuclear SRF expressivity in bone and soft tissues suggests SRF has a mechanistic role in bone metastasis.

Immuno-histochemical characterisation of a man’s disease necessitates a biopsy specimen. Although this is not the current standard of care for prostate cancer patients, biopsy of new lesions in other malignancies has led to treatment adjustments being carried out in as few as one in seven patients [55]. Indeed in the context of prostate cancer, despite its multifocal and multi-clonal heterogeneity, most distant metastases from different anatomic sites in the same patient share the majority of genetic alterations [56–60]. As there is an increased risk of bone fracture amongst this population, where Melton et al. noted that 58% of men with castration resistant prostate cancer sustain at least 1 pathologic fracture [61], fixation of such fractures could represent one suitable time-point to obtain a biopsy for immuno-histochemical analysis. Surgery has remained the dominant modality by which solid cancers have been sampled for such analyses, and some note that metastatic tissue is often inaccessible and the purity and yield of biopsy samples are low [62]. More recently though, work by the Michigan Oncology Sequencing Project (MI-ONCOSEQ) [63], Hong et al. in Melbourne [64], and Van Allen et al.[65] have successfully demonstrated that with improved techniques and tools, the vehicle by which metastatic tissue will be obtained for a model of personalised medicine is image-guided percutaneous biopsy.

In order to investigate the functional role of SRF we undertook SRF siRNA knockdown experiments and demonstrated significant reversal in resistance to docetaxel in our PC-3 model of docetaxel resistant prostate cancer. Previous studies by Prencipe et al. have demonstrated that in a LNCaP model of castration resistance, that SRF inhibition impacts upon cell death and proliferation [6]. As mentioned earlier, studies of the role of SRF in prostate cancer are limited. However Taylor et al. have demonstrated that SRF inhibition leads to integrin activation and trafficking, and so reduces migration of neutrophils in response to inflammation in both in-vivo and in-vitro studies [66]. Knockout of SRF reduces Enigma; a LIM domain protein which has been shown to be highly expressed in bone metastases and may function as an oncoprotein [67]. Coupled together these findings further suggest that SRF may play a role in progression of prostate cancer, and maybe an amenable therapeutic target for manipulation at various disease stages [6, 50].

There are limitations to the present study. Because the functional work was performed in a single cell line it is difficult to make absolute assumptions about the generalizability of the finding that resistance to docetaxel can be overcome by inhibiting SRF in men with advanced metastatic prostate cancer. Nevertheless, in a personalised medicine approach where each man’s disease is appropriately profiled, SRF inhibition may form part of an appropriate therapeutic pathway.

## Conclusions

The diagnosis and treatment of prostate cancer is based on a series of clinical, radiological and pathological criteria. Implicit in the adaptive and progressive nature of the disease is that the biological characteristics and sensitivity of metastases to hormonal and chemotherapies are likely to change from that of the primary lesion; and so further characterisation of metastatic lesions will be required. Identification of treatment resistant prostate cancer which is ultimately the most lethal form of the disease is a great clinical challenge. Stratification of prostate cancer at an early stage could reduce the overtreatment of many prostate cancers, identify those who are most likely to progress sooner, and help predict response to hormone and chemotherapies. Such markers to stratify the significance of these prostate cancers at an early stage are notably absent from the literature. Our results suggest that SRF could be one such marker, and may also represent a therapeutic target in the treatment of men afflicted with advanced prostate cancer.

## Additional files

Additional file 1: Table S1. Characteristics of patient cohort and differences between Docetaxel naïve and Docetaxel resistant patients. *N* = 42. Values are presented as mean (standard deviation). * *P*-values <0.05 indicate if there is statistically significant difference between the groups characteristics (Docetaxel naïve vs. Docetaxel resistant). (DOCX 19 kb)
Additional file 2: Table S2. Genes predicted to by dysregulated in the studied PC3 model of Docetaxel Resistance. AG = aged matched control, D8 = docetaxel resistant subline, D12 = docetaxel resistant subline. FC = fold change. (DOCX 79 kb)

## Abbreviations

Ag: Age matched controls
ATCC: American Type Culture Collection
BGA: Between group analysis
CIA: Co-Inertia Analysis
D8 and D12: PC-3 resistant sublines
ESR1: Oestrogen-receptor 1
HSF1: Heat shock factor protein 1
IHC: Immunohistochemical
mCDRPC: Metastatic castration and docetaxel resistant prostate cancer
mCRPC: Metastatic castration resistant prostate cancer
MI-ONCOSEQ: Michigan Oncology Sequencing Project
MTT: 3-(4,5)-dimethylthiazol-2-yl-2,5-diphenyltetrazolium bromide
NFκB: Nuclear factor kappa-B
OS: Overall survival
PI3K: Phosphatidylinositol 3′-kinase
SRF: Serum response factor
TFBS: Transcription factor binding site
TFs: Transcription factors
TR2 &4: Testicular receptor 2 & 4
VDR-RXR: Vitamin-D and retinoid x receptor
