# Supplementary material for: The prognostic utility of the transcription factor SRF in docetaxel-resistant prostate cancer: in-vitro discovery and in-vivo validation
Source: BMC Cancer. 2017 Mar 1;17:163. doi: 10.1186/s12885-017-3100-4 (PMC5333466; doi:10.1186/s12885-017-3100-4)
Supplement: Additional file 1: Table S1. — Characteristics of patient cohort and differences between Docetaxel naïve and Docetaxel resistant patients. N = 42. Values are presented as mean (standard deviation). * P-values <0.05 indicate if there is statistically significant difference between the groups characteristics (Docetaxel naïve vs. Docetaxel resistant). (DOCX 19 kb) [file 12885_2017_3100_MOESM1_ESM.docx]

|  | Docetaxel Naïve (N=19) | Docetaxel Resistant (N=23) | All Patients (N=42) | P-Value (*) |
| --- | --- | --- | --- | --- |
| Age (Years) | 65.18 (8.63) | 62.4 (10.08) | 63.86 (9.34) | 0.34 |
| PSA at diagnosis (ng/ml) | 424 (1109) | 118.2 (174.9) | 260 (766) | 0.3 |
| Gleason Score | 7.8 (1.2) | 7.4 (1.3) | 7.6 (1.26) | 0.28 |
| Survival from Diagnosis (Years) | 8.2 (5.58) | 5.511 (3.75) | 6.9 (4.9) | 0.077 |
| Survival from AI (Years) | 2.714 (2.043) | 2.241 (1.553) | 2.4 (1.8) | 0.45 |
| Survival from Bone Metastasis (Years) | 2.536 (2.118) | 7.6 (22.04) | 4.9 (1.2) | 0.3 |

Supplemental Table 1: Characteristics of patient cohort and differences between Docetaxel naïve and Docetaxel resistant patients. N=42. Values are presented as mean (standard deviation). * P-values <0.05 indicate if there is statistically significant difference between the groups characteristics (Docetaxel naïve vs. Docetaxel resistant).
