# Supplementary material for: The prognostic utility of the transcription factor SRF in docetaxel-resistant prostate cancer: in-vitro discovery and in-vivo validation
Source: BMC Cancer. 2017 Mar 1;17:163. doi: 10.1186/s12885-017-3100-4 (PMC5333466; doi:10.1186/s12885-017-3100-4)
Supplement: Additional file 2: Table S2. — Genes predicted to by dysregulated in the studied PC3 model of Docetaxel Resistance. AG = aged matched control, D8 = docetaxel resistant subline, D12 = docetaxel resistant subline. FC = fold change. (DOCX 79 kb) [file 12885_2017_3100_MOESM2_ESM.docx]

Supplemental Table 2: Genes predicted to by dysregulated in the studied PC3 model of Docetaxel Resistance. AG= aged matched control, D8 = docetaxel resistant subline, D12=docetaxel resistant subline. FC= fold change.

|  |  |  | D8 vs AG | | D12 vs AG | |
| --- | --- | --- | --- | --- | --- | --- |
| Gene | **Full Name** | **Genetic locus** | **Log FC** | **Adjusted P-value** | **Log FC** | **Adjusted P-value** |
| ABCB8 | ATP-binding cassette, sub-family B (MDR/TAP), member 8 | 7q36 | -0.487008176 | 0.001111541 | -0.581066897 | 0.001392508 |
| ABHD2 | abhydrolase domain containing 2 | 15q26.1 | -0.503663093 | 0.000244912 | -0.635952358 | 0.000301714 |
| ACBD7 | acyl-CoA binding domain containing 7 | 10p13 | -0.430764146 | 0.040215514 | -1.210689173 | 0.000696665 |
| ADAMTS20 | ADAM metallopeptidase with thrombospondin type 1 motif, 20 | 12q12 | -0.39374566 | 0.010519615 | -0.508766806 | 0.007007381 |
| AMOT | angiomotin | Xq23 | -0.864074292 | 0.000239785 | -1.351673483 | 9.41E-05 |
| ANGPTL4 | angiopoietin-like 4 | 19p13.3 | 0.552078189 | 0.003840682 | 0.678975052 | 0.003527841 |
| ANKRD36 | ankyrin repeat domain 36 | 2q11.2 | 0.405721204 | 0.007706697 | 0.553588595 | 0.00404016 |
| ANKRD36B | ankyrin repeat domain 36B | 2q11.2 | 0.981678384 | 0.000152436 | 1.170613994 | 0.000269686 |
| ANKRD37 | ankyrin repeat domain 37 | 4q35.1 | -0.468001812 | 0.006861845 | -0.471091645 | 0.015016561 |
| ANO2 | anoctamin 2 | 12p13.3 | -0.546615967 | 0.039559915 | -0.61121074 | 0.047403433 |
| ANO6 | anoctamin 6 | 12q12 | -0.451899857 | 0.035322892 | -0.537231827 | 0.033508263 |
| AP1S1 | adaptor-related protein complex 1, sigma 1 subunit | 7q22.1 | -0.440273986 | 0.001412519 | -0.87563019 | 0.000132391 |
| AREG | amphiregulin | 4q13-q21 | 0.561853718 | 0.000339805 | 0.667162829 | 0.000541568 |
| ARF5 | ADP-ribosylation factor 5 | 7q31.3 | -0.686840463 | 0.002497151 | -0.751246551 | 0.004211681 |
| ARID3B | AT rich interactive domain 3B (BRIGHT-like) | 15q24 | -0.680629873 | 0.003858562 | -0.762401738 | 0.005454092 |
| ASB1 | ankyrin repeat and SOCS box-containing 1 | 2q37 | 0.385658596 | 0.00262351 | 0.549508831 | 0.001216405 |
| ASB13 | ankyrin repeat and SOCS box-containing 13 | 10p15.1 | -0.931807051 | 0.000304165 | -1.098128491 | 0.000514512 |
| AZGP1 | alpha-2-glycoprotein 1, zinc-binding | 7q22.1 | -1.745044197 | 0.031471045 | -1.913136845 | 0.041394854 |
| B3GALNT1 | beta-1,3-N-acetylgalactosaminyltransferase 1 (globoside blood group) | 3q25 | 0.416050954 | 0.024012781 | 0.650459783 | 0.006527164 |
| BCKDK | branched chain ketoacid dehydrogenase kinase | 16p11.2 | 0.43144131 | 0.00284286 | 0.433234859 | 0.007082866 |
| BCL7B | B-cell CLL/lymphoma 7B | 7q11.23 | -0.512407642 | 0.007072099 | -0.629042481 | 0.006082185 |
| BCL9L | B-cell CLL/lymphoma 9-like | 11q23.3 | -0.55133142 | 0.010268777 | -0.584317337 | 0.01725312 |
| BFAR | bifunctional apoptosis regulator | 16p13.12 | 0.435083389 | 0.004270351 | 0.552597063 | 0.003328632 |
| BNIP3 | BCL2/adenovirus E1B 19kDa interacting protein 3 | 10q26.3 | 0.623190612 | 0.006356058 | 0.666445669 | 0.010682178 |
| BTBD11 | BTB (POZ) domain containing 11 | 12q23.3 | 0.805425769 | 8.72E-05 | 1.001136414 | 0.000137372 |
| C10orf18 | chromosome 10 open reading frame 18 | 10p15.1 | -0.458051623 | 0.004100535 | -0.650208091 | 0.001884453 |
| C10orf25 | chromosome 10 open reading frame 25 | 10q11.21 | -0.824299377 | 0.000105429 | -0.840124891 | 0.000440714 |
| C11orf17 | chromosome 11 open reading frame 17 | 11p15.3 | -0.40788906 | 0.002096657 | -0.996135914 | 6.30E-05 |
| C11orf41 | chromosome 11 open reading frame 41 | 11p13 | 1.168241683 | 6.45E-05 | 1.182596597 | 0.000306313 |
| C15orf38 | chromosome 15 open reading frame 38 | 15q26.1 | -0.450877701 | 0.034897355 | -0.570508602 | 0.025654743 |
| C16orf52 | chromosome 16 open reading frame 52 | 16p12.2 | 0.553559002 | 0.007130518 | 0.8120431 | 0.002629626 |
| C16orf53 | chromosome 16 open reading frame 53 | 16p11.2 | 0.459754325 | 0.038884794 | 0.718603465 | 0.011268704 |
| C18orf21 | chromosome 18 open reading frame 21 | 18q12.2 | -0.464214308 | 0.005870747 | -0.48525523 | 0.01103819 |
| C1orf21 | chromosome 1 open reading frame 21 | 1q25 | 0.866415498 | 1.66E-05 | 1.133160262 | 2.45E-05 |
| C2CD2L | C2CD2-like | 11q23.3 | -0.530054931 | 0.000400967 | -0.80157897 | 0.000181173 |
| C3orf26 | chromosome 3 open reading frame 26 | 3q12.1 | -0.784564016 | 0.00012367 | -1.109312479 | 9.25E-05 |
| C4orf41 | chromosome 4 open reading frame 41 | 4q35.1 | -0.444753897 | 0.003046012 | -0.498694086 | 0.004467538 |
| C4orf43 | chromosome 4 open reading frame 43 | 4q32.2 | -0.387069683 | 0.009125426 | -0.439993387 | 0.011212339 |
| C5orf13 | chromosome 5 open reading frame 13 | 5q22.1 | 0.381426758 | 0.005392902 | 0.707170985 | 0.000651516 |
| C5orf44 | chromosome 5 open reading frame 44 | 5q12.3 | -0.467208714 | 0.006074529 | -0.649175492 | 0.002956238 |
| C5orf54 | chromosome 5 open reading frame 54 | 5q33.3 | 0.679632371 | 0.002961275 | 0.796890469 | 0.003529837 |
| C7orf41 | chromosome 7 open reading frame 41 | 7p14.3 | -0.485020281 | 0.013805728 | -0.615674517 | 0.009941402 |
| C7orf46 | chromosome 7 open reading frame 46 | 7p15.3 | -0.544000856 | 0.000526983 | -0.97703265 | 9.25E-05 |
| C7orf58 | chromosome 7 open reading frame 58 | 7q31.31 | -0.614684678 | 0.009161698 | -0.787661001 | 0.006335342 |
| C7orf64 | chromosome 7 open reading frame 64 | 7q21.2 | -0.596753779 | 0.000126633 | -0.618100156 | 0.000477478 |
| C9orf72 | chromosome 9 open reading frame 72 | 9p21.2 | 0.449558475 | 0.003898359 | 0.507284017 | 0.005330511 |
| CABLES1 | Cdk5 and Abl enzyme substrate 1 | 18q11.2 | 0.677604116 | 0.00106484 | 0.845452261 | 0.00105972 |
| CAPRIN2 | caprin family member 2 | 12p11 | 0.948287566 | 5.62E-06 | 0.990509873 | 3.03E-05 |
| CARD16 | caspase recruitment domain family, member 16 | --- | -0.618765241 | 0.004313602 | -0.797003681 | 0.003140076 |
| CD97 | CD97 molecule | 19p13 | -0.4092453 | 0.002248973 | -0.579999797 | 0.001076247 |
| CDC16 | cell division cycle 16 homolog (S. cerevisiae) | 13q34 | -0.666736413 | 0.000134077 | -0.848455969 | 0.000173933 |
| CDK15 | cyclin-dependent kinase 15 | 2q33.2 | -0.480904945 | 0.001003449 | -0.50158901 | 0.002459185 |
| CEACAM6 | carcinoembryonic antigen-related cell adhesion molecule 6 (non-specific cross reacting antigen) | 19q13.2 | 1.352302619 | 0.000955406 | 2.142921005 | 0.000304225 |
| CHCHD2 | coiled-coil-helix-coiled-coil-helix domain containing 2 | 7p11.2 | -0.486391032 | 0.008346829 | -0.587150194 | 0.007739314 |
| CHPF | chondroitin polymerizing factor | 2q35 | -0.726207038 | 3.19E-05 | -0.875803861 | 6.46E-05 |
| CNTNAP3 | contactin associated protein-like 3 | 9p13.1 | 1.783919867 | 3.46E-05 | 2.180269619 | 6.46E-05 |
| COBLL1 | COBL-like 1 | 2q24.3 | -0.954944943 | 0.000148586 | -1.105270292 | 0.000306999 |
| COL4A5 | collagen, type IV, alpha 5 | Xq22 | 0.560361599 | 0.00032952 | 0.935407501 | 8.98E-05 |
| COL4A6 | collagen, type IV, alpha 6 | Xq22 | 0.81524223 | 4.52E-05 | 0.99034753 | 8.59E-05 |
| CREG1 | cellular repressor of E1A-stimulated genes 1 | 1q24 | -0.416443512 | 0.003267843 | -0.423233136 | 0.007521731 |
| CTDSP2 | CTD (carboxy-terminal domain, RNA polymerase II, polypeptide A) small phosphatase 2 | 12q14.1 | -0.474812105 | 0.00261952 | -0.663139499 | 0.001354649 |
| CUX1 | cut-like homeobox 1 | 7q22.1 | -0.427292777 | 0.014213253 | -0.458716322 | 0.021806965 |
| CXCL16 | chemokine (C-X-C motif) ligand 16 | 17p13 | -0.745670918 | 0.016332113 | -0.817436249 | 0.022599273 |
| CYP4F11 | cytochrome P450, family 4, subfamily F, polypeptide 11 | 19p13.1 | -0.696633597 | 0.049058961 | -0.905931758 | 0.032496661 |
| DFNA5 | deafness, autosomal dominant 5 | 7p15 | -0.518901294 | 0.021054102 | -0.660608212 | 0.014902452 |
| DLG1 | discs, large homolog 1 (Drosophila) | 3q29 | -0.580287592 | 0.002114039 | -0.602876699 | 0.004711127 |
| DNAJC12 | DnaJ (Hsp40) homolog, subfamily C, member 12 | 10q22.1 | -1.093341527 | 8.24E-05 | -1.488530587 | 8.02E-05 |
| DNAJC2 | DnaJ (Hsp40) homolog, subfamily C, member 2 | 7q22 | -0.447880165 | 0.010273828 | -0.503560737 | 0.013166999 |
| DTX3L | deltex 3-like (Drosophila) | 3q21.1 | -0.911799789 | 3.56E-05 | -0.912833465 | 0.000193696 |
| DUSP23 | dual specificity phosphatase 23 | 1q23.2 | -0.522031297 | 0.00140801 | -0.628769703 | 0.001644869 |
| ECHDC1 | enoyl CoA hydratase domain containing 1 | 6q22.33 | -0.495156857 | 0.037557364 | -0.621118667 | 0.028693119 |
| EDNRA | endothelin receptor type A | 4q31.22 | 0.640627859 | 0.00368487 | 1.584092262 | 0.000101893 |
| EIF4A2 | eukaryotic translation initiation factor 4A2 | 3q28 | -0.58905612 | 0.007523042 | -0.607919054 | 0.014587507 |
| ELMO3 | engulfment and cell motility 3 | 16q22.1 | -0.469278425 | 0.001818233 | -0.484116478 | 0.004265032 |
| EMR2 | egf-like module containing, mucin-like, hormone receptor-like 2 | 19p13.1 | -0.720694109 | 0.000397821 | -0.74711737 | 0.00117419 |
| EPHB3 | EPH receptor B3 | 3q21-qter | -0.390220088 | 0.009439661 | -0.597860424 | 0.00279164 |
| EPHB6 | EPH receptor B6 | 7q33-q35 | 0.482942363 | 0.000872337 | 0.644672197 | 0.00065751 |
| EPRS | glutamyl-prolyl-tRNA synthetase | 1q41 | -0.539891891 | 0.002623604 | -0.626604074 | 0.003328632 |
| ERCC4 | excision repair cross-complementing rodent repair deficiency, complementation group 4 | 16p13.12 | 0.810255551 | 0.000109703 | 0.887451329 | 0.000316252 |
| ETFA | electron-transfer-flavoprotein, alpha polypeptide | 15q23-q25 | -0.390138743 | 0.000700059 | -0.510069475 | 0.000610306 |
| EYA4 | eyes absent homolog 4 (Drosophila) | 6q23 | -0.529051118 | 0.000930493 | -0.532972596 | 0.002721772 |
| FAM102B | family with sequence similarity 102, member B | 1p13.3 | 0.727258252 | 0.000477613 | 0.982282967 | 0.000378748 |
| FAM129A | family with sequence similarity 129, member A | 1q25 | -1.225574086 | 2.90E-05 | -1.792906549 | 2.25E-05 |
| FAM46A | family with sequence similarity 46, member A | 6q14 | 0.659517996 | 0.000121757 | 0.719255135 | 0.000352833 |
| FBXO18 | F-box protein, helicase, 18 | 10p15.1 | -0.56847198 | 0.001997288 | -0.744639552 | 0.001475059 |
| FMNL2 | formin-like 2 | 2q23.3 | 0.520535827 | 0.013560359 | 0.570329317 | 0.019286182 |
| FOXF1 | forkhead box F1 | 16q24 | -0.474849823 | 0.012786504 | -0.497000475 | 0.022240369 |
| FUCA1 | fucosidase, alpha-L- 1, tissue | 1p34 | -0.412968638 | 0.005988733 | -0.430115801 | 0.011421098 |
| GDPD5 | glycerophosphodiester phosphodiesterase domain containing 5 | 11q13.4-q13.5 | -0.458712482 | 0.003996824 | -0.528560736 | 0.004949034 |
| GFPT2 | glutamine-fructose-6-phosphate transaminase 2 | 5q34-q35 | 0.504564251 | 0.005092467 | 0.900739225 | 0.000733302 |
| GLI3 | GLI family zinc finger 3 | 7p13 | 1.264561924 | 1.24E-05 | 1.713514622 | 1.62E-05 |
| GMPR | guanosine monophosphate reductase | 6p23 | -0.51261144 | 0.001100618 | -0.544727646 | 0.002429605 |
| GNB2 | guanine nucleotide binding protein (G protein), beta polypeptide 2 | 7q21.3-q22.1\|7q22 | -0.417426074 | 0.003511029 | -0.464839317 | 0.005186407 |
| GOLGA8IP | golgin A8 family, member I (pseudogene) | 15q11.2 | 0.506488832 | 0.00094514 | 0.545252258 | 0.00201019 |
| GPR155 | G protein-coupled receptor 155 | 2q31.1 | 0.993483245 | 1.07E-05 | 1.011210108 | 6.05E-05 |
| GPR18 | G protein-coupled receptor 18 | 13q32 | -0.408127483 | 0.001288014 | -0.444161888 | 0.002459185 |
| GPR64 | G protein-coupled receptor 64 | Xp22.13 | 0.904903078 | 8.05E-05 | 0.904996988 | 0.000390008 |
| GSTA4 | glutathione S-transferase alpha 4 | 6p12.1 | 0.403837205 | 0.000685305 | 0.483649145 | 0.000897708 |
| GSTO2 | glutathione S-transferase omega 2 | 10q25.1 | -0.700061916 | 0.003709607 | -0.809519236 | 0.004573203 |
| GTF3C1 | general transcription factor IIIC, polypeptide 1, alpha 220kDa | 16p12 | 0.410582324 | 0.015857643 | 0.471155608 | 0.018122022 |
| GTPBP4 | GTP binding protein 4 | 10p15-p14 | -0.634107831 | 4.95E-05 | -0.78168993 | 8.59E-05 |
| HEG1 | HEG homolog 1 (zebrafish) | 3q21.2 | -1.650340751 | 0.003579134 | -2.100055006 | 0.00279164 |
| HEPH | hephaestin | Xq11-q12 | -0.451079952 | 0.003938087 | -0.538043024 | 0.004158552 |
| HKR1 | HKR1, GLI-Kruppel zinc finger family member | 19q13.12 | 0.628710813 | 0.00535036 | 0.90507232 | 0.002226853 |
| HLA-DPA1 | major histocompatibility complex, class II, DP alpha 1 | 6p21.3 | -1.510016142 | 0.000742644 | -1.901332117 | 0.00076139 |
| HOMER1 | homer homolog 1 (Drosophila) | 5q14.2 | 0.385479402 | 0.005407061 | 0.542622746 | 0.002485106 |
| HSP90AA1 | heat shock protein 90kDa alpha (cytosolic), class A member 1 | 14q32.33 | 0.417960402 | 0.000866765 | 0.506479169 | 0.001037751 |
| HSP90AA2 | heat shock protein 90kDa alpha (cytosolic), class A member 2 | 11p14.1 | 0.616433322 | 8.32E-05 | 0.689437536 | 0.000220967 |
| HSP90AA4P | heat shock protein 90kDa alpha (cytosolic), class A member 4 (pseudogene) | 4q35.2 | 0.541432171 | 0.00095354 | 0.594730306 | 0.001844697 |
| IFITM3 | interferon induced transmembrane protein 3 (1-8U) | 11p15.5 | -0.490743817 | 0.007833019 | -0.996707731 | 0.000601008 |
| IFRD1 | interferon-related developmental regulator 1 | 7q31.1 | -0.674160821 | 0.000130705 | -0.703318905 | 0.000471353 |
| IGF1R | insulin-like growth factor 1 receptor | 15q26.3 | -0.585124404 | 0.028595746 | -0.944378211 | 0.006792731 |
| IL15 | interleukin 15 | 4q31 | -0.726471748 | 0.001662918 | -0.852214978 | 0.002141337 |
| IL1RL1 | interleukin 1 receptor-like 1 | 2q12 | 0.981986202 | 0.007401669 | 2.230831799 | 0.000316252 |
| IL6 | interleukin 6 (interferon, beta 2) | 7p21 | -0.974874091 | 9.54E-05 | -1.154278215 | 0.000189278 |
| IL6ST | interleukin 6 signal transducer (gp130, oncostatin M receptor) | 5q11 | -0.432908798 | 0.011571261 | -0.475430958 | 0.016415187 |
| ING3 | inhibitor of growth family, member 3 | 7q31 | -0.61323046 | 0.000960345 | -1.028867997 | 0.000222252 |
| IRF1 | interferon regulatory factor 1 | 5q31.1 | -0.531919921 | 0.006164067 | -0.655258238 | 0.005288821 |
| ITPR1 | inositol 1,4,5-triphosphate receptor, type 1 | 3p26.1 | 0.651305366 | 5.42E-05 | 0.982679216 | 3.15E-05 |
| JAK2 | Janus kinase 2 | 9p24 | 0.600688633 | 0.000520595 | 1.732674891 | 9.63E-06 |
| KCNJ2 | potassium inwardly-rectifying channel, subfamily J, member 2 | 17q24.3 | 0.801581333 | 0.000206303 | 0.841406262 | 0.000655195 |
| KCNN4 | potassium intermediate/small conductance calcium-activated channel, subfamily N, member 4 | 19q13.2 | -0.453809321 | 0.003718364 | -0.751476524 | 0.000802671 |
| KDM1B | lysine (K)-specific demethylase 1B | 6p22.3 | -0.380178166 | 0.003704466 | -0.422928149 | 0.005439426 |
| KIAA0226 | KIAA0226 | 3q29 | -0.383386152 | 0.000823761 | -0.664337504 | 0.000167403 |
| KIAA1432 | KIAA1432 | 9p24.1 | 0.441357743 | 0.003507444 | 0.585636242 | 0.002271646 |
| KIAA2018 | KIAA2018 | 3q13.2 | -0.716823426 | 0.001320532 | -0.853297172 | 0.001646955 |
| KIF3C | kinesin family member 3C | 2p23 | -0.389545191 | 0.003539565 | -0.514410744 | 0.002342704 |
| KIN | KIN, antigenic determinant of recA protein homolog (mouse) | 10p15-p14 | -0.546083468 | 0.004286865 | -0.889229885 | 0.000984467 |
| KLF6 | Kruppel-like factor 6 | 10p15 | -0.946458088 | 3.47E-05 | -1.031737505 | 0.000125494 |
| KLF7 | Kruppel-like factor 7 (ubiquitous) | 2q32 | -0.730514782 | 0.000185813 | -0.73722209 | 0.000724593 |
| KLHDC10 | kelch domain containing 10 | 7q32.2 | -0.602512618 | 0.004486246 | -0.891448087 | 0.001681175 |
| KLHL35 | kelch-like 35 (Drosophila) | 11q13.4 | -0.957418209 | 1.77E-05 | -1.019428441 | 7.71E-05 |
| KMO | kynurenine 3-monooxygenase (kynurenine 3-hydroxylase) | 1q42-q44 | -1.161573463 | 0.03950676 | -1.441430835 | 0.031446274 |
| KRT32 | keratin 32 | 17q21.2 | -0.389281635 | 0.024420611 | -0.502562941 | 0.016374035 |
| KRT80 | keratin 80 | 12q13.13 | -0.913120828 | 8.54E-05 | -1.042818756 | 0.000205917 |
| LARP4B | La ribonucleoprotein domain family, member 4B | 10p15.3 | -0.726128007 | 0.000145421 | -0.751337846 | 0.000534475 |
| LAT2 | linker for activation of T cells family, member 2 | 7q11.23 | -0.423569669 | 0.006042447 | -0.426470136 | 0.0134533 |
| LATS1 | LATS, large tumor suppressor, homolog 1 (Drosophila) | 6q25.1 | -0.407341788 | 0.001251823 | -0.458596629 | 0.002045565 |
| LGALS9 | lectin, galactoside-binding, soluble, 9 | 17q11.2 | -0.642055948 | 0.035712422 | -0.751888708 | 0.036057451 |
| LINGO2 | leucine rich repeat and Ig domain containing 2 | 9p21.2 | 0.588124805 | 0.016154293 | 1.03566306 | 0.002454599 |
| LMBR1L | limb region 1 homolog (mouse)-like | 12q13.12 | -0.483278417 | 0.028112664 | -0.615838826 | 0.020050991 |
| LOC387790 | hypothetical LOC387790 | 11q13.5 | 0.477390645 | 0.007591788 | 0.518428252 | 0.011625549 |
| LOXL4 | lysyl oxidase-like 4 | 10q24 | -1.603917151 | 0.011786243 | -1.64987214 | 0.022249752 |
| LPHN1 | latrophilin 1 | 19p13.2 | -0.633570018 | 0.000170757 | -0.668406122 | 0.00054944 |
| LRCH2 | leucine-rich repeats and calponin homology (CH) domain containing 2 | Xq23 | 1.250355274 | 2.99E-05 | 2.045978976 | 1.54E-05 |
| LRCH4 | leucine-rich repeats and calponin homology (CH) domain containing 4 | 7q22 | -0.42969882 | 0.044538644 | -0.556539965 | 0.029895295 |
| LRRC6 | leucine rich repeat containing 6 | 8q24.22 | 0.641871968 | 0.005020019 | 2.252366799 | 2.25E-05 |
| LRRC61 | leucine rich repeat containing 61 | 7q31-q35 | -0.409089794 | 0.011747836 | -0.502385179 | 0.009936926 |
| LRRK1 | leucine-rich repeat kinase 1 | 15q26.3 | -0.414923181 | 0.008808771 | -0.467076189 | 0.01133663 |
| MAGI1 | membrane associated guanylate kinase, WW and PDZ domain containing 1 | 3p14.1 | 0.445400547 | 0.021216304 | 0.529803263 | 0.020475957 |
| MAN2A2 | mannosidase, alpha, class 2A, member 2 | 15q26.1 | -0.590126705 | 0.032407823 | -0.83751864 | 0.014272302 |
| MBD6 | methyl-CpG binding domain protein 6 | --- | -0.416080381 | 0.024298562 | -0.632524421 | 0.007558369 |
| MBL1P | mannose-binding lectin (protein A) 1, pseudogene | 10q22.2-q22.3 | -0.429996764 | 0.005482776 | -0.47646868 | 0.007863298 |
| MEGF9 | multiple EGF-like-domains 9 | 9q32-q33.3 | 0.716007587 | 5.09E-05 | 0.732587637 | 0.000235822 |
| METTL7B | methyltransferase like 7B | 12q13.2 | -0.586462417 | 0.018813803 | -1.380140532 | 0.000696741 |
| MINA | MYC induced nuclear antigen | 3q11.2 | -0.414994832 | 0.000541919 | -0.732247153 | 0.000102655 |
| MMP17 | matrix metallopeptidase 17 (membrane-inserted) | 12q24.3 | -0.496396634 | 0.001326075 | -0.537475253 | 0.00257641 |
| MRPS24 | mitochondrial ribosomal protein S24 | 7p14 | -0.468953623 | 0.000840852 | -0.50573379 | 0.001820612 |
| MTHFD1L | methylenetetrahydrofolate dehydrogenase (NADP+ dependent) 1-like | 6q25.1 | -0.405583544 | 0.005250162 | -0.418608129 | 0.010605595 |
| MTMR8 | myotubularin related protein 8 | Xq11.2 | -0.908347958 | 0.002289862 | -1.304362193 | 0.001039096 |
| MUC3A | mucin 3A, cell surface associated | 7q22 | -0.676176616 | 0.002540234 | -0.834401649 | 0.00241683 |
| MYLK | myosin light chain kinase | 3q21 | -1.197705682 | 0.000339878 | -1.236297219 | 0.001047924 |
| NDE1 | nudE nuclear distribution gene E homolog 1 (A. nidulans) | 16p13.11 | 0.477134063 | 0.000579733 | 0.484216841 | 0.001807672 |
| NDUFA4 | NADH dehydrogenase (ubiquinone) 1 alpha subcomplex, 4, 9kDa | 7p21.3 | -0.428509782 | 0.005247409 | -0.441583092 | 0.010682178 |
| NEDD4 | neural precursor cell expressed, developmentally down-regulated 4 | 15q | -0.452184388 | 0.003933068 | -0.713728559 | 0.001052173 |
| NET1 | neuroepithelial cell transforming 1 | 10p15 | -0.540936793 | 9.84E-05 | -0.576843238 | 0.000332618 |
| NFATC2IP | nuclear factor of activated T-cells, cytoplasmic, calcineurin-dependent 2 interacting protein | 16p11.2 | 0.524548529 | 0.026052649 | 0.539310555 | 0.044639022 |
| NFKB2 | nuclear factor of kappa light polypeptide gene enhancer in B-cells 2 | 10q24 | -0.417086677 | 0.006929036 | -0.7693676 | 0.000829646 |
| NIT2 | nitrilase family, member 2 | 3q12.2 | -0.582161326 | 6.94E-05 | -0.582497515 | 0.000343384 |
| NOVA1 | neuro-oncological ventral antigen 1 | 14q | 1.162409747 | 8.10E-06 | 2.089519797 | 3.58E-06 |
| NQO1 | NAD(P)H dehydrogenase, quinone 1 | 16q22.1 | 0.432491557 | 0.0058943 | 0.588624415 | 0.003174427 |
| NRIP1 | nuclear receptor interacting protein 1 | 21q11.2 | 0.39779923 | 0.015908196 | 0.405277203 | 0.030035787 |
| NSUN6 | NOP2/Sun domain family, member 6 | 10p12.31 | 0.429911263 | 0.009756126 | 0.446083598 | 0.018144795 |
| NUAK1 | NUAK family, SNF1-like kinase, 1 | 12q23.3 | -0.514472262 | 0.008778895 | -0.697389096 | 0.004711127 |
| NUDT5 | nudix (nucleoside diphosphate linked moiety X)-type motif 5 | 10p14 | -0.708014941 | 0.001320532 | -0.930137172 | 0.000988166 |
| OPTN | optineurin | 10p13 | -0.722382804 | 0.001176716 | -0.949433532 | 0.000894127 |
| OR2T8 | olfactory receptor, family 2, subfamily T, member 8 | 1q44 | -0.459366727 | 0.025263038 | -0.549403706 | 0.02355346 |
| P2RX7 | purinergic receptor P2X, ligand-gated ion channel, 7 | 12q24 | -1.588572076 | 0.00065347 | -1.956610761 | 0.00076139 |
| PAK1 | p21 protein (Cdc42/Rac)-activated kinase 1 | 11q13-q14 | -0.498660107 | 0.001570027 | -1.364230181 | 2.55E-05 |
| PALMD | palmdelphin | 1p22-p21 | 1.030931796 | 0.001505902 | 3.009792561 | 1.90E-05 |
| PDAP1 | PDGFA associated protein 1 | 7q22.1 | -0.50373027 | 0.001778657 | -0.569130855 | 0.002703941 |
| PDE4DIP | phosphodiesterase 4D interacting protein | 1q12 | -0.417772884 | 0.01962453 | -0.482913751 | 0.021401634 |
| PDP1 | pyruvate dehyrogenase phosphatase catalytic subunit 1 | 8q22.1 | -0.85123066 | 0.001878338 | -0.864629587 | 0.004737379 |
| PFKM | phosphofructokinase, muscle | 12q13.3 | -0.523529024 | 0.002830522 | -0.878121579 | 0.000601008 |
| PHLDB2 | pleckstrin homology-like domain, family B, member 2 | 3q13.2 | -1.147191624 | 4.14E-06 | -1.85223236 | 3.58E-06 |
| PIGB | phosphatidylinositol glycan anchor biosynthesis, class B | 15q21-q22 | -0.397232064 | 0.002707748 | -0.762072708 | 0.000293042 |
| PIGK | phosphatidylinositol glycan anchor biosynthesis, class K | 1p31.1 | 0.572215973 | 0.007327357 | 0.801646796 | 0.003394849 |
| PIKFYVE | phosphoinositide kinase, FYVE finger containing | 2q34 | -0.40908621 | 0.005678219 | -0.518474755 | 0.004299641 |
| PITRM1 | pitrilysin metallopeptidase 1 | 10p15.2 | -0.518751899 | 0.005785534 | -0.716863475 | 0.00289357 |
| PLA2G16 | phospholipase A2, group XVI | 11q12.3 | -0.537003335 | 0.001601474 | -0.653269257 | 0.001756638 |
| PML | promyelocytic leukemia | 15q22 | -0.493725122 | 0.00186587 | -0.747547271 | 0.000678005 |
| PNPLA8 | patatin-like phospholipase domain containing 8 | 7q31 | -0.567025265 | 0.000697938 | -0.82703659 | 0.000352833 |
| PODXL | podocalyxin-like | 7q32-q33 | -0.925086216 | 2.26E-05 | -1.176542498 | 3.65E-05 |
| PPWD1 | peptidylprolyl isomerase domain and WD repeat containing 1 | 5q12.3 | -0.647798599 | 0.000634462 | -0.735868519 | 0.001082529 |
| PRDM1 | PR domain containing 1, with ZNF domain | 6q21 | -1.770897834 | 5.03E-06 | -2.11763911 | 1.54E-05 |
| PRDX5 | peroxiredoxin 5 | 11q13 | -0.546620335 | 0.000400008 | -0.600533419 | 0.000885476 |
| PREX1 | phosphatidylinositol-3,4,5-trisphosphate-dependent Rac exchange factor 1 | 20q13.13 | 0.549354899 | 0.01907439 | 0.760800026 | 0.009166088 |
| PRKAA2 | protein kinase, AMP-activated, alpha 2 catalytic subunit | 1p31 | 0.864294292 | 0.000159285 | 1.079516699 | 0.000217671 |
| PRKAG2 | protein kinase, AMP-activated, gamma 2 non-catalytic subunit | 7q36.1 | -0.426045789 | 0.008842874 | -0.436542784 | 0.017559062 |
| PROS1 | protein S (alpha) | 3q11.2 | -0.576000912 | 0.009959933 | -0.762953213 | 0.005878824 |
| PSMB9 | proteasome (prosome, macropain) subunit, beta type, 9 (large multifunctional peptidase 2) | 6p21.3 | -1.073999495 | 8.04E-06 | -1.377705561 | 1.54E-05 |
| PTER | phosphotriesterase related | 10p12 | -0.46485153 | 0.000903809 | -0.90861032 | 9.41E-05 |
| PTGFR | prostaglandin F receptor (FP) | 1p31.1 | 0.864795094 | 0.000210076 | 1.714014624 | 2.45E-05 |
| PTK7 | PTK7 protein tyrosine kinase 7 | 6p21.1-p12.2 | 0.447815924 | 0.002481951 | 0.461352618 | 0.00553808 |
| PTPRK | protein tyrosine phosphatase, receptor type, K | 6q22.2-q22.3 | -0.392507027 | 0.001948519 | -0.687609655 | 0.000343384 |
| PYGB | phosphorylase, glycogen; brain | 20p11.2-p11.1 | 0.395188761 | 0.044695796 | 0.482118181 | 0.038129202 |
| RAB23 | RAB23, member RAS oncogene family | 6p11 | 0.523965761 | 0.017446738 | 0.673745097 | 0.011746899 |
| RAB5B | RAB5B, member RAS oncogene family | 12q13 | -0.676666661 | 0.002703219 | -0.698734195 | 0.005915439 |
| RAD51L1 | RAD51-like 1 (S. cerevisiae) | 14q23-q24.2 | 0.678741221 | 8.05E-05 | 0.888640073 | 9.41E-05 |
| RAET1L | retinoic acid early transcript 1L | 6q25.1 | -0.783631056 | 0.005012479 | -0.791894514 | 0.011245131 |
| RDH11 | retinol dehydrogenase 11 (all-trans/9-cis/11-cis) | 14q24.1 | 0.487825447 | 0.001101364 | 0.610900126 | 0.001064127 |
| REPIN1 | replication initiator 1 | 7q36.1 | -0.535143879 | 0.002174069 | -0.613427966 | 0.003005322 |
| RGL1 | ral guanine nucleotide dissociation stimulator-like 1 | 1q25.3 | 0.534861303 | 0.001016921 | 0.891340647 | 0.000246851 |
| RIPK2 | receptor-interacting serine-threonine kinase 2 | 8q21 | -0.380741426 | 0.004713389 | -0.438180199 | 0.005737896 |
| RMRP | RNA component of mitochondrial RNA processing endoribonuclease | 9p21-p12 | -0.45580053 | 0.039251304 | -1.173690724 | 0.001027979 |
| RNF169 | ring finger protein 169 | 11q13.4 | -0.393204736 | 0.008032797 | -0.442652311 | 0.010410352 |
| RNF216 | ring finger protein 216 | 7p22.1 | -0.392127266 | 0.004220307 | -0.396468141 | 0.009727869 |
| RPL22L1 | ribosomal protein L22-like 1 | 3q26.2 | -0.498597988 | 0.003617311 | -0.742198664 | 0.001324277 |
| RPL24 | ribosomal protein L24 | 3q12 | -0.387016451 | 0.01094804 | -0.639389355 | 0.002272784 |
| RPP38 | ribonuclease P/MRP 38kDa subunit | 10p13 | -0.590372435 | 0.022461834 | -0.689527107 | 0.023351778 |
| RPS12 | ribosomal protein S12 | 6q23.2 | -0.456655552 | 0.000749158 | -0.523489123 | 0.001211679 |
| RSL24D1 | ribosomal L24 domain containing 1 | 15q21 | -0.50295287 | 0.027424795 | -0.535045737 | 0.040985048 |
| SCAMP2 | secretory carrier membrane protein 2 | 15q23-q25 | -0.462669416 | 0.000294095 | -0.621710597 | 0.00025301 |
| SCNN1G | sodium channel, nonvoltage-gated 1, gamma | 16p12 | 0.617155801 | 0.014441431 | 1.551695057 | 0.000382331 |
| SEMA7A | semaphorin 7A, GPI membrane anchor (John Milton Hagen blood group) | 15q22.3-q23 | -0.452030017 | 0.003384775 | -0.995133011 | 0.000177153 |
| SERAC1 | serine active site containing 1 | 6q25.3 | -0.552163709 | 0.002985879 | -0.702376633 | 0.002409655 |
| SFT2D1 | SFT2 domain containing 1 | 6q27 | -0.56567629 | 0.000893961 | -0.566329174 | 0.002717596 |
| SIPA1L3 | signal-induced proliferation-associated 1 like 3 | 19q13.13 | -0.387649372 | 0.032247434 | -0.445935632 | 0.035083183 |
| SLC25A21 | solute carrier family 25 (mitochondrial oxodicarboxylate carrier), member 21 | 14q11.2 | 0.953308356 | 0.002453913 | 1.365299024 | 0.001109526 |
| SLC4A11 | solute carrier family 4, sodium borate transporter, member 11 | 20p12 | 1.313790497 | 0.000130896 | 1.432900761 | 0.000378748 |
| SLC4A4 | solute carrier family 4, sodium bicarbonate cotransporter, member 4 | 4q21 | 1.077667544 | 1.39E-05 | 1.768135765 | 8.57E-06 |
| SLCO4C1 | solute carrier organic anion transporter family, member 4C1 | 5q21.2 | -0.532987609 | 0.02789429 | -0.65825947 | 0.02263764 |
| SLFN12L | schlafen family member 12-like | 17q12 | -0.537158223 | 0.04025778 | -0.618930074 | 0.043033686 |
| SMAD3 | SMAD family member 3 | 15q22.33 | -0.589931269 | 0.000799446 | -0.739544557 | 0.000826574 |
| SMARCAL1 | SWI/SNF related, matrix associated, actin dependent regulator of chromatin, subfamily a-like 1 | 2q35 | -0.426600426 | 0.024095505 | -0.566146117 | 0.014157211 |
| SNORA20 | small nucleolar RNA, H/ACA box 20 | 6q25.3 | -0.507917002 | 0.002909088 | -0.536205967 | 0.005674048 |
| SNORA45 | small nucleolar RNA, H/ACA box 45 | 11p15.4 | -0.702727434 | 0.001409488 | -0.756597881 | 0.00278877 |
| SNORD115-11 | small nucleolar RNA, C/D box 115-11 | 15q11.2 | -0.402385401 | 0.005997192 | -0.457247733 | 0.007571379 |
| SNRPA1 | small nuclear ribonucleoprotein polypeptide A' | 15q26.3 | -0.621709385 | 0.003542642 | -0.664411923 | 0.006333078 |
| SOBP | sine oculis binding protein homolog (Drosophila) | 6q21 | 1.033912113 | 0.000360889 | 1.041328159 | 0.001254354 |
| SPIN4 | spindlin family, member 4 | Xq11.1 | 0.393914939 | 0.004070462 | 0.584717966 | 0.001503528 |
| SPPL2A | signal peptide peptidase-like 2A | 15q21.2 | -0.432119295 | 0.027105978 | -0.493433197 | 0.030550088 |
| SSH3 | slingshot homolog 3 (Drosophila) | 11q13.2 | -0.445563777 | 0.004027303 | -0.472940269 | 0.007351679 |
| SYVN1 | synovial apoptosis inhibitor 1, synoviolin | 11q13 | -0.398019094 | 0.012404059 | -0.582956801 | 0.00455865 |
| TAF3 | TAF3 RNA polymerase II, TATA box binding protein (TBP)-associated factor, 140kDa | 10p15.1 | -0.437790141 | 0.001206778 | -0.827212731 | 0.000148821 |
| TAF5L | TAF5-like RNA polymerase II, p300/CBP-associated factor (PCAF)-associated factor, 65kDa | 1q42.13 | -0.507706152 | 0.000376683 | -0.515296229 | 0.001248901 |
| TAF6 | TAF6 RNA polymerase II, TATA box binding protein (TBP)-associated factor, 80kDa | 7q22.1 | -0.546431814 | 0.001897771 | -0.61621225 | 0.002891429 |
| TAF7 | TAF7 RNA polymerase II, TATA box binding protein (TBP)-associated factor, 55kDa | 5q31 | -0.499113174 | 0.000113731 | -0.593140242 | 0.000213166 |
| TAS2R14 | taste receptor, type 2, member 14 | 12p13 | 0.551599917 | 0.01030035 | 0.619022273 | 0.013304929 |
| TAS2R50 | taste receptor, type 2, member 50 | 12p13.2 | 0.539494331 | 0.006888461 | 0.675550407 | 0.005378395 |
| TBC1D23 | TBC1 domain family, member 23 | 3q12.2 | -0.647861232 | 0.000634127 | -1.029052066 | 0.000206906 |
| TFG | TRK-fused gene | 3q12.2 | -0.392190987 | 0.002851368 | -0.605520489 | 0.000885476 |
| TGM4 | transglutaminase 4 (prostate) | 3p22-p21.33 | -0.424861039 | 0.002294967 | -0.427688053 | 0.00580322 |
| THOC6 | THO complex 6 homolog (Drosophila) | 16p13.3 | 0.476159663 | 0.000554065 | 0.80984986 | 0.000131326 |
| TM2D3 | TM2 domain containing 3 | 15q26.3 | -0.454617402 | 0.001207006 | -0.680635494 | 0.000496817 |
| TMBIM1 | transmembrane BAX inhibitor motif containing 1 | 2p24.3-p24.1 | -0.742687639 | 1.96E-05 | -2.587830597 | 1.75E-07 |
| TMED3 | transmembrane emp24 protein transport domain containing 3 | 15q24-q25 | -0.571949653 | 0.000984897 | -0.590652723 | 0.002544473 |
| TMEM168 | transmembrane protein 168 | 7q31.32 | -0.395788329 | 0.017553829 | -0.972304709 | 0.000530502 |
| TMEM2 | transmembrane protein 2 | 9q13-q21 | 0.718696397 | 3.85E-05 | 1.256263089 | 1.35E-05 |
| TMEM80 | transmembrane protein 80 | 11p15.5 | -0.41111558 | 0.003298626 | -0.73505533 | 0.000502109 |
| TMEM9B | TMEM9 domain family, member B | 11p15.3 | -0.534115411 | 0.00353334 | -0.707433816 | 0.00230271 |
| TMPRSS11E | transmembrane protease, serine 11E | 4q13.2 | -0.41590778 | 0.006516218 | -0.453065927 | 0.010005596 |
| TMPRSS15 | transmembrane protease, serine 15 | 21q21\|21q21.1 | -0.435867472 | 0.028581865 | -0.482768483 | 0.036372452 |
| TNFAIP3 | tumor necrosis factor, alpha-induced protein 3 | 6q23 | -0.432235485 | 0.004265841 | -0.536911259 | 0.003689033 |
| TNFRSF14 | tumor necrosis factor receptor superfamily, member 14 (herpesvirus entry mediator) | 1p36.32 | -0.493340999 | 0.024765765 | -0.872949387 | 0.00375904 |
| TOB2 | transducer of ERBB2, 2 | 22q13.2 | -0.463779833 | 0.029393932 | -0.653119689 | 0.01332458 |
| TOX | thymocyte selection-associated high mobility group box | 8q12.1 | 0.993085467 | 5.09E-05 | 1.00727392 | 0.000247554 |
| TPK1 | thiamin pyrophosphokinase 1 | 7q34-q35 | -0.456396194 | 0.018433352 | -0.883473168 | 0.001804039 |
| TRIM2 | tripartite motif-containing 2 | 4q31.3 | -1.173804986 | 2.03E-05 | -1.40724168 | 4.51E-05 |
| TRIM21 | tripartite motif-containing 21 | 11p15.5 | -0.496015548 | 0.006136471 | -0.717385973 | 0.002459185 |
| TRIM64 | tripartite motif-containing 64 | 11q14.3 | -0.436058188 | 0.000460333 | -0.573447718 | 0.000416623 |
| TRIP11 | thyroid hormone receptor interactor 11 | 14q31-q32 | 0.427627544 | 0.025788092 | 0.653254541 | 0.00788403 |
| TRPM7 | transient receptor potential cation channel, subfamily M, member 7 | 15q21 | -0.413248326 | 0.002392753 | -0.443624679 | 0.004467372 |
| TTC6 | tetratricopeptide repeat domain 6 | 14q21.1 | 0.581039882 | 0.017813899 | 1.321985141 | 0.000773722 |
| TTLL4 | tubulin tyrosine ligase-like family, member 4 | 2p24.3-p24.1 | -0.658469895 | 4.40E-05 | -0.661248547 | 0.000227763 |
| TYMS | thymidylate synthetase | 18p11.32 | 0.45867898 | 0.02414233 | 0.487267385 | 0.036867365 |
| UBA7 | ubiquitin-like modifier activating enzyme 7 | 3p21 | -1.205583213 | 0.023281368 | -1.303478813 | 0.033096973 |
| UCP2 | uncoupling protein 2 (mitochondrial, proton carrier) | 11q13 | -0.557021147 | 0.000487735 | -1.233757342 | 2.77E-05 |
| UFSP2 | UFM1-specific peptidase 2 | 4q35.1 | -0.471301699 | 0.000535898 | -0.546637123 | 0.000864621 |
| UGDH | UDP-glucose 6-dehydrogenase | 4p15.1 | 0.734393119 | 5.38E-05 | 0.852456127 | 0.000131326 |
| ULBP2 | UL16 binding protein 2 | 6q25 | -0.659687482 | 0.004776704 | -0.756271953 | 0.005915439 |
| UPF2 | UPF2 regulator of nonsense transcripts homolog (yeast) | 10p14-p13 | -0.494413537 | 0.002565304 | -0.746876842 | 0.000890772 |
| UQCRC2 | ubiquinol-cytochrome c reductase core protein II | 16p12 | 0.38112258 | 0.002755488 | 0.478764639 | 0.002387225 |
| USP6NL | USP6 N-terminal like | 10p13 | -0.53554079 | 0.005544252 | -0.747321228 | 0.002639939 |
| VRK1 | vaccinia related kinase 1 | 14q32 | 0.439382968 | 0.046338326 | 1.022728868 | 0.002098879 |
| VSIG1 | V-set and immunoglobulin domain containing 1 | Xq22.3 | 3.028734861 | 2.42E-06 | 3.185187414 | 1.54E-05 |
| VSNL1 | visinin-like 1 | 2p24.3 | -0.386188194 | 0.035281677 | -0.531936003 | 0.018085034 |
| WDR54 | WD repeat domain 54 | 2p13.1 | 0.470008428 | 0.026170007 | 0.538972106 | 0.029130447 |
| WDR61 | WD repeat domain 61 | 15q25.1 | -0.385718778 | 0.000971603 | -0.571893925 | 0.000427728 |
| WT1 | Wilms tumor 1 | 11p13 | 0.750775189 | 0.006851742 | 0.899865258 | 0.006628764 |
| YKT6 | YKT6 v-SNARE homolog (S. cerevisiae) | 7p15.1 | -0.381042565 | 0.006270073 | -0.430694158 | 0.008072473 |
| ZBTB24 | zinc finger and BTB domain containing 24 | 6q21 | -0.419999169 | 0.001751853 | -0.62303926 | 0.000708623 |
| ZDBF2 | zinc finger, DBF-type containing 2 | 2q33.3 | -0.577721701 | 0.024771085 | -1.995020653 | 0.000138152 |
| ZFYVE1 | zinc finger, FYVE domain containing 1 | 14q24.2 | -0.521661043 | 0.005312624 | -0.589021312 | 0.007008696 |
| ZNF148 | zinc finger protein 148 | 3q21 | -0.448611349 | 0.009418409 | -0.727221916 | 0.002141337 |
| ZNF277 | zinc finger protein 277 | 7q31.1 | -0.413917738 | 0.012236144 | -0.461204288 | 0.016226079 |
| ZNF33B | zinc finger protein 33B | 10q11.2 | -0.484325021 | 0.008230264 | -0.885676065 | 0.001027474 |
| ZNF398 | zinc finger protein 398 | 7q36.1 | -0.467542192 | 0.001800417 | -0.640472955 | 0.001052858 |
| ZNF592 | zinc finger protein 592 | 15q25.3 | -0.413149902 | 0.00077079 | -0.426606494 | 0.002091487 |
| ZNF774 | zinc finger protein 774 | 15q26.1 | -0.515592402 | 0.001215585 | -0.548416356 | 0.002614359 |
| ZNF841 | zinc finger protein 841 | 19q13.41 | -1.042117399 | 9.89E-05 | -1.538802456 | 5.89E-05 |
|  |  |  |  |  |  |  |
